# Supplementary figures and images for: Implementing an intervention to enhance care delivery and consistency for people with hip fracture and cognitive impairment in acute hospital wards: a mixed methods process evaluation of a randomised controlled feasibility trial (PERFECTED)
Source: BMJ Open. 2023 Feb 3;13(2):e064482. doi: 10.1136/bmjopen-2022-064482 (PMC9900060; doi:10.1136/bmjopen-2022-064482)

Supplementary Figure 1: PERFECT-ER logic model

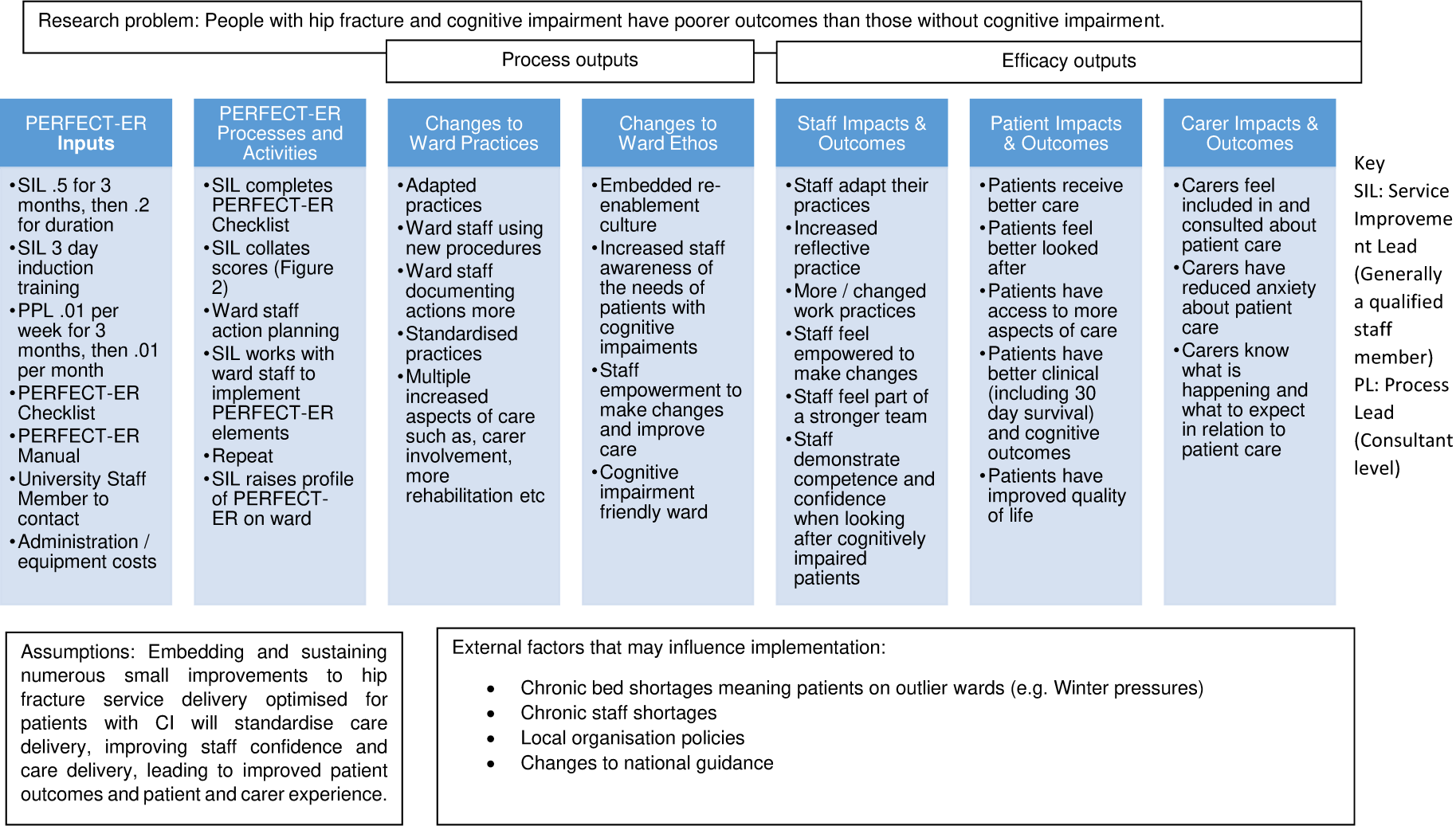

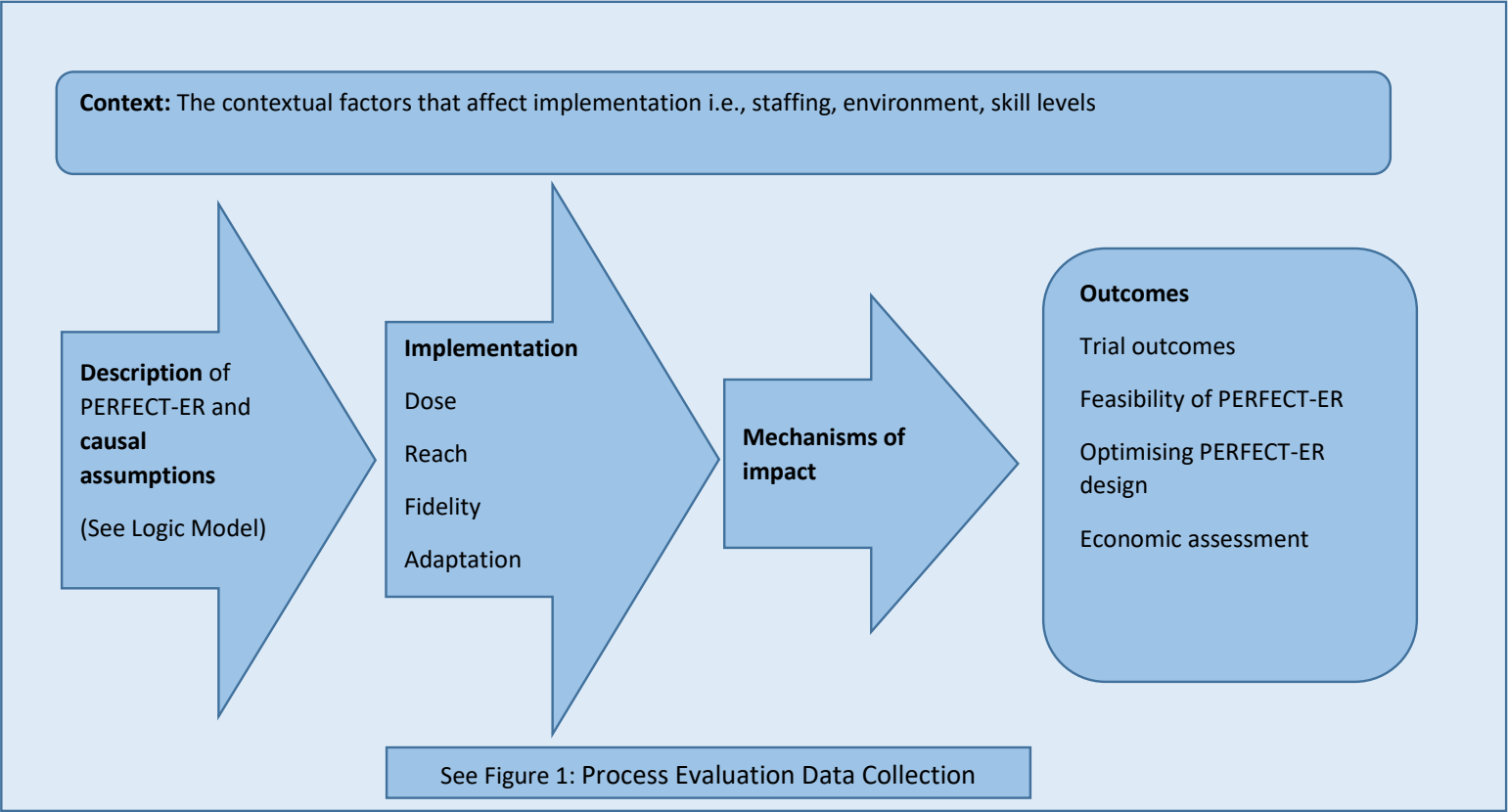

Supplement: Supplementary data [file bmjopen-2022-064482supp001.pdf]
