# Supplementary material for: Implementing an intervention to enhance care delivery and consistency for people with hip fracture and cognitive impairment in acute hospital wards: a mixed methods process evaluation of a randomised controlled feasibility trial (PERFECTED)
Source: BMJ Open. 2023 Feb 3;13(2):e064482. doi: 10.1136/bmjopen-2022-064482 (PMC9900060; doi:10.1136/bmjopen-2022-064482)
Supplement: Author's manuscript [file bmjopen-2022-064482.draft_revisions.pdf]

BMJ Open is committed to open peer review. As part of this commitment we make the peer review history of every article we publish publicly available.

When an article is published we post the peer reviewers' comments and the authors' responses online. We also post the versions of the paper that were used during peer review. These are the versions that the peer review comments apply to.

The versions of the paper that follow are the versions that were submitted during the peer review process. They are not the versions of record or the final published versions. They should not be cited or distributed as the published version of this manuscript.

BMJ Open is an open access journal and the full, final, typeset and author-corrected version of record of the manuscript is available on our site with no access controls, subscription charges or pay-per-view fees (http://bmjopen.bmj.com).

If you have any questions on BMJ Open's open peer review process please email
info.bmjopen@bmj.com

BMJ Open

Implementing an intervention to enhance care delivery and consistency for people with hip fracture and cognitive impairment in acute hospital wards: a mixed methods process evaluation of a randomised controlled feasibility trial (PERFECTED)

Journal:	*BMJ Open*
Manuscript ID	bmjopen-2022-064482
Article Type:	Original research
Date Submitted by the Author:	17-May-2022

Complete List of Authors:	Backhouse, Tamara; University of East Anglia, School of Health Sciences<br>Fox, Chris; Norwich Medical School, Department of Psychological Sciences; University of Exeter,<br>Hammond, Simon; University of East Anglia, Norwich Medical School<br>Poland, Fiona; University of East Anglia, School of Health Sciences<br>McDermott-Thompson, Vicki; University of East Anglia<br>Penhale, Bridget; University of East Anglia<br>Cross, Jane; University of East Anglia

Keywords:	Dementia < NEUROLOGY, Delirium & cognitive disorders < PSYCHIATRY, ORTHOPAEDIC & TRAUMA SURGERY

SCHOLARONE™
Manuscripts

I, the Submitting Author has the right to grant and does grant on behalf of all authors of the Work (as defined in the below author licence), an exclusive licence and/or a non-exclusive licence for contributions from authors who are: i) UK Crown employees; ii) where BMJ has agreed a CC-BY licence shall apply, and/or iii) in accordance with the terms applicable for US Federal Government officers or employees acting as part of their official duties; on a worldwide, perpetual, irrevocable, royalty-free basis to BMJ Publishing Group Ltd ("BMJ") its licensees and where the relevant Journal is co-owned by BMJ to the co-owners of the Journal, to publish the Work in this journal and any other BMJ products and to exploit all rights, as set out in our licence.

The Submitting Author accepts and understands that any supply made under these terms is made by BMJ to the Submitting Author unless you are acting as an employee on behalf of your employer or a postgraduate student of an affiliated institution which is paying any applicable article publishing charge ("APC") for Open Access articles. Where the Submitting Author wishes to make the Work available on an Open Access basis (and intends to pay the relevant APC), the terms of reuse of such Open Access shall be governed by a Creative Commons licence – details of these licences and which Creative Commons licence will apply to this Work are set out in our licence referred to above.

Other than as permitted in any relevant BMJ Author's Self Archiving Policies, I confirm this Work has not been accepted for publication elsewhere, is not being considered for publication elsewhere and does not duplicate material already published. I confirm all authors consent to publication of this Work and authorise the granting of this licence.

For peer review only

BMJ OPEN TITLE PAGE

Title: Implementing an intervention to enhance care delivery and consistency for people with hip fracture and cognitive impairment in acute hospital wards: a mixed methods process evaluation of a randomised controlled feasibility trial (PERFECTED)

Authors and Affiliations:

Tamara Backhouse[1] Chris Fox[2] Simon P Hammond[3] Fiona Poland[1] Vicki McDermott-Thompson[4] Bridget Penhale[1] Jane L Cross[1]*

1 School of Health Sciences, University of East Anglia, Norwich Research Park, Norwich, UK

2 College of Medicine and Health, University of Exeter, Exeter, EX1 2HZ, UK

3 School of Education & Lifelong Learning, University of East Anglia, Norwich Research Park, Norwich, UK

4 School of Psychology, University of East Anglia, Norwich Research Park, Norwich, UK

***Corresponding author's contact details:**

Dr Jane L. Cross

Email: j.cross@uea.ac.uk

Key words: Cognitive impairment, Dementia, hip fracture, Implementation, acute hospital, Process evaluation

Manuscript 3888words

Abstract 295 words

Tables 1

Figures 2

Supplementary tables 1

For peer review only - http://bmjopen.bmj.com/site/about/guidelines.xhtml

For peer review only

STRUCTURED ABSTRACT:

Objectives: To determine how, and under what circumstances, the PERFECT-ER intervention was implemented in five acute hospital wards and impacted on staff practices and perceptions.

Design: Mixed methods process evaluation (undertaken between 2016-18).

Setting: Five acute hospital wards across three different UK regions.

Participants: Patients (n=3) admitted to acute wards with hip fracture and cognitive impairment, their relatives (n=29) and hospital staff (n=63).

Interventions: PERFECT-ER, a multi-component intervention designed to enhance the recovery of patients with hip fracture and cognitive impairment was implemented for 18-months. PERFECT-ER was implemented at ward level ensuring that multiple new and existing practices were undertaken consistently, on the assumption that collectively, small individual advances would improve care delivery for patients.

Primary and secondary outcome measures: Implementation of the PERFECT-ER intervention examined through regular intervention scores, service improvement staff reports and action plans, and semi-structured interviews and focus groups.

Results: The process evaluation identified points of implementation vulnerability and strength. All wards implemented some elements of PERFECT-ER. Implementation was fragile when ward pressures were high and when ward staff perceived the relative priority of intervention practices to be low. Adaptations to the implementation process may have reduced whole-ward staff engagement with implementation. However, strategic enlistment of senior ward influencers (such as ward managers, orthogeriatricians) combined with service improvement lead in-ward peer pressure tactics facilitated implementation processes.

Conclusions: Our study suggests that implementation was expediated when existing and new power structures were used as mechanisms of impact. Within hierarchical settings such as acute wards, key individuals appeared to influence implementation through endorsement and sometimes enforcement. This indicates that whole-ward interventions may not always require cognitive engagement from all ward staff to implement changes. Future ward-level

For peer review only

implementation studies could consider how best to engage staff and most importantly, which staff to best target.

Trial registration: ISRCTN99336264

Strengths and limitations:

- Strengths of this study include obtaining data from multiple sources and stakeholders from five different acute hospital wards across seven implementation cycles.
- Another strength was the independence of the core process evaluation team from the feasibility trial team.
- Due to the nature of the intervention, documentary evidence from patients' notes was used by improvement leads to complete intervention checklists and this information could not be verified by researchers.
- The research team could not confirm whether implementation was due to changes in clinical care, documenting practices or due to improvement leads' own actions.

Funding statement: This work was supported by NIHR Programme Grants for Applied Research (PGfAR) Programme grant number [ref: DTC-RP-PG-0311-12004].

Ethical Approval: Ethical approval for the trial was given by Camden and Kings Research Ethics Committee (reference number: 16/LO/0621) and Scotland Research Ethics Committee A (reference number: 16/SS/0086).

Competing interests statement: The authors declare they have no competing interests.

For peer review only

Background

The increasing incidence of dementia is a world-wide public health challenge, requiring multiple strategies including action to improve the quality of healthcare services[1]. Providing acute care for patients with cognitive impairment (including those with either diagnosed or assumed dementia and/or delirium) is challenging for hospital staff[2,3]. Patients with cognitive impairment have complex needs including delirium, behaviours that challenge, incontinence, dehydration, and communication difficulties. These make necessary medical, nursing and rehabilitation processes more difficult for staff to undertake[2,4-7].

In acute settings, over 40% of people with a hip fracture have cognitive impairment[8]. Usual biomedical approaches[9] and limited evidence for rehabilitation for these patients[10-12] create further difficulties for staff. Consequently, patients with hip fracture and cognitive impairment have increased risk of unfavourable outcomes[13-16], impaired rehabilitation and recovery[6,17], with a worse prognosis than patients without cognitive impairment[8,18]. Hospital staff need to manage these complexities and complex multifaceted interventions to improve quality in healthcare delivery, and outcomes are increasingly required[19].

The PERFECTED research programme was funded by The National Institute for Health Research (NIHR), UK, to develop and test an intervention to enhance the recovery of patients with hip fracture and cognitive impairment on acute wards. PERFECT-ER is a complex multicomponent service improvement intervention which aims to enhance recovery using multiple marginal gains in the quality of care[20-23]. Marginal gains include the implementation of new practices, standardisation of existing practices, ensuring consistency, and cultivating a whole-ward 'in-it-together' ethos.

The PERFECT-ER intervention comprises a structured checklist, Service Improvement Lead (SIL), Process Lead (PL) and implementation manual using a Plan, Do, Study, Act (PDSA) approach to facilitate behaviour change[24-25]see logic model (Supplementary table 1). PERFECT-ER is a whole ward approach involving all staff delivering patient care. The PERFECT-ER checklist has 68 practice items (including clinical, functional, psychosocial, and cognitive elements), which span admission, pre-operative, post-operative, rehabilitation, and discharge periods and 15 organisational items, which highlight the policies behind current practices in the hospital. Checklists have been used before to improve the quality of care[26,27], however

For peer review only - http://bmjopen.bmj.com/site/about/guidelines.xhtml

such lists rarely change practice unless combined with mechanisms for change within the socio-practice contexts of hospital settings[28,29]. For example, much relational work in hospitals relies on individual staff members and supportive organisational structures[30].

PERFECT-ER is implemented by a SIL, a formally appointed internal implementation lead, a qualified healthcare professional, based day-to-day on the selected ward. They attend a three-day induction training in intervention delivery where they were provided with the intervention checklist and a comprehensive instruction manual. They were supported by a senior doctor acting as a PL to facilitate implementation across the organisation. The checklist, designed to overlay current hip fracture pathways, identifies areas of strength and weaknesses in current practice. It is used to score a sample of patients' notes (documentary evidence) to determine current practice and identify areas for improvement. Scores are shared with staff at action-planning meetings, where discussions create shared action plans to address lower scoring items. The Plan (action plan), Do (implementation), Study (fill in checklist and assess scores), Act (engage ward staff) cycle then recommences.

As part of the wider PERFECTED programme of research, we conducted a process evaluation of the PERFECT-ER feasibility trial[20,23]. The aim of this process evaluation was to determine how and under what circumstances the PERFECT-ER intervention was implemented in five acute hospital wards and impacted on staff practices and perceptions (for published protocol, please see supplementary material) [20,31-33]. Qualitative data from process evaluations of trials are widely used to generate understandings of intervention implementation[34] and can be of critical importance[35]. However, there is limited qualitative evidence about the implementation of complex interventions to enhance recovery in hospital settings[36]. Therefore, to examine implementation in acute wards in detail, we used a mixed methods approach incorporating qualitative data.

Ethical approval for the PERFECTED feasibility trial and process evaluation was granted by _____ ____ ____ Research Ethics Committee ([date], reference). The trial was registered with ISRCTN [number].

METHODS

The process evaluation took place in five different acute NHS hospital wards in five separate geographical areas across England and Scotland where the PERFECT-ER intervention was

For peer review only - http://bmjopen.bmj.com/site/about/guidelines.xhtml

being implemented. Resource use and patient and relative outcomes are reported elsewhere[23]. We used the Medical Research Council guidance[37-39] for the evaluation and the Standards for Reporting Implementation Studies (StaRI)[40].

Process evaluation data were gathered between August 2016 and March 2018 over seven PDSA cycles (see Figure 1 for data collected from each ward). We collected PERFECT-ER checklist scores, SIL-generated reports and action plans, and semi-structured interview and focus group data from each ward. PERFECT-ER checklist scores were obtained from patient notes by the SILs. At each ward there were eight checklist time-points: 3 months pre-trial, 1.5 months pre-trial, trial baseline, 4 months, 7 months, 10 months, 13 months, and end of trial (15 months). Written data comprised SILs' regular action plans outlining the items identified to implement each cycle and the progress made in relation to these, and reflective reports listing the actions taken day-to-day when implementing the items in the action plans.

Figure 1 here

We undertook: semi-structured face-to-face or telephone interviews with hospital staff and relatives of patients; focus groups with hospital staff; and face-to-face interviews with patients. We purposively sampled ward-based staff (up to 15 from each ward) who had been in contact with the intervention. Interviews and focus groups with staff investigated their knowledge of, and engagement with, the intervention and implementation processes. These were undertaken after trial processes finished to prevent interviews impacting on implementation. Patients and relatives were offered the opportunity to take part in interviews by a research nurse during a follow-up visit for the trial (5 patients and 5 relatives from each ward). Interviews were designed to explore patients and relatives' experiences of the intervention ward. All had capacity to consent. Patients and relatives' interviews took place in their home or in a private room at the hospital according to their wishes. All interviews and focus groups were conducted by academic researchers not collecting data or consent for the feasibility trial (SPH, TB and VM-T).

Patient and public involvement

For peer review only - http://bmjopen.bmj.com/site/about/guidelines.xhtml

PERFECTED prioritised Public patient involvement (PPI) from grant development to completion. PPI representatives provided feedback on participant information materials and the study protocol, and had roles on the Trial Steering Committee, Data Monitoring Ethics Committee, and Advisory Groups. For the process evaluation, PPI carer representatives co-interviewed, with researchers, in six interviews with relatives. They received training and support to facilitate this.

Analysis

All interviews and focus groups were digitally recorded and transcribed verbatim. Identifying features were removed to ensure confidentiality and anonymity. Qualitative analysis was iterative throughout the data collection and thematic, focussing on implementation processes informed by the MRC Process Evaluations of Complex Interventions[37-39] including dose, reach, fidelity, adaptations, mechanisms of impact, and context and The Consolidated Framework for Implementation Research (CFIR)[41]. NVivo software was used to manage the data. TB generated initial codes from repeated reading of data, identifying tentative themes, before multiple analysis meetings with the study team (JLC, SPH, FP, VM-T, CF), which refined and developed these analyses both across sites and within individual sites. Quantitative data from PERFECT-ER checklist scores were analysed descriptively by ward and implementation cycle.

Results

Eighty-one interviews and focus groups with 95 participants, were conducted, Table 1 describes this population.

Table 1: Participant Demographics

Participant	Total n=95 *n*(%)	Female *n*(%)	Age Mean (range)	Method of data collection by *n*		
				Face-to-face interview	Telephone interview	Focus Group
Patient	3(3)	3(100)	78(68-87)	3	0	0
Relatives*	29(31)	22(76)	57(22-75)	7	22	0
Service Improvement Lead	6(6)	5(83)	45(29-59)	1	5	0

For peer review only - http://bmjopen.bmj.com/site/about/guidelines.xhtml

Process Lead (Consultant Geriatricians)	5(5)	1(20)	44 (37-49)	0	5	0
Research Nurse	8(8)	8(100)	43(26-54)	0	6	2
Hospital staff**	44(46)	43(98)	39(22-60)	19	1	24

**daughter (n=15), son (n=3), daughter-in-law (n=4), son-in-law (n=3), niece (n=1), granddaughter (n=1), husband (n=1), sister-in-law (n=1)*

***nurse (n=12), senior nurse (n=9), healthcare assistant (n=8), occupational health therapist (n=4), physiotherapist (n=3), ward administrator (n=2), ward housekeeper (n=2), doctor (n=1), trauma assistant (n=1), general therapy assistant (n=1), clinical trial assistant (n=1)*

For peer review only

Dose

Figure 2 shows PERFECT-ER checklist scores for each ward by percentage of elements delivered throughout the study. All wards implemented some elements of PERFECT-ER. Dose varied across wards, with some implementing multiple items and others struggling or having fluctuating results. Contextual factors appeared to help or hinder implementation. Wards B and C implemented well; these sites had dedicated SIL time and clear ward processes which could be utilised for implementation. Wards A, D and E struggled with severe ward pressures affecting staff practices and wards D and E had difficulties providing dedicated SIL time and a lack of consistent support from ward staff. Ward D had a different SIL obtain the baseline data from the rest of the cycles. These initial scores were 16% higher than any other ward, perhaps because this individual scored more positively than the other SILs. Average dose delivered was 68%.

Figure 2 here

Reach

A proportion of ward staff at all sites engaged with PERFECT-ER to some degree. Many staff at Ward C were largely unaware of the study and when asked about changes due to the intervention their typical response was:

For peer review only - http://bmjopen.bmj.com/site/about/guidelines.xhtml

Nurse Practitioner: *I wouldn't have thought, not through PERFECT-ER no, I wouldn't think so no* (Ward C)

Staff Nurse: *Or if it is through PERFECT-ER no-one has told us it has come from that* (Ward C)

Nevertheless, PERFECT-ER elements implemented for the study were now part of their usual daily practice and valued.

…all the tools that we are bringing in are certainly useful to help appropriately nurse the patients that we actually do have (Nurse Practitioner, Ward C)

This suggests that implementation of PERFECT-ER elements did not rely on the engagement of all ward staff with the process.

In wards C and E several staff members said they would have liked more information about the intervention and an active role with implementation.

Just to know more about it [PERFECT-ER] *really… So we could get more involved basically.* (Physiotherapist, Ward E)

This suggests optimal reach was not always achieved, nevertheless elements of PERFECT-ER were viewed as having improved staff awareness, particularly of pain and delirium:

It's brought an awareness of delirium because a lot of the time in the past people would say 'oh that's behaviour' and maybe just put it down to dementia type behaviour (Occupational Therapist, Ward C)

Interviews with relatives and patients suggested they did not notice an enhanced care experience. They expressed mixed experiences of care generally, but no-one noted PERFECT-ER specifically in their interview.

By and large as I say it was a good experience umm I'd, I had done it before… I think it was better than it was four years ago (Relative, Ward C)

We felt like we were being a negative complaining family in a way because things just weren't going right (Relative, Ward A)

For peer review only - http://bmjopen.bmj.com/site/about/guidelines.xhtml

Indicators of implementation may have reflected changes in documentary practices, and not changes in clinical practice as staff noted modifications to standardise recording and assessment practices.

> *I don't think it changed my practice… it helped standardise those that perhaps wouldn't routinely do the same thing, but for those that were doing it, it allowed us to be clearly documented* (PL, Ward E)

Fidelity

Many aspects of PERFECT-ER were implemented as intended. However, the intervention was developed with one SIL intended as the appointed implementation leader. However, two wards employed three individuals to conduct the SIL role between them.

> *At times I was only being allocated one 11.5 hour shift for PERFECT-ER in 8-12 weeks periods. Once this was raised to my management team, they tried to give an hour or two on occasional days …subject to how busy the ward was so found that majority of the times this ended up not being used* (SIL Report, Ward D)

> *I do not feel I have been very supported by the other SILs …due to workload on the project ward and lack of staff* (SIL, Ward E)

In these wards, SIL time was not protected and teamwork between SILs was lacking, often with one SIL taking on the full work with little support and reduced time. Both wards (D & E) had drops in implementation (see Figure 2).

Two SILs from these wards (D and E) changed their own practices by ensuring PERFECT-ER documents were present in patients' notes instead of encouraging ward practices to change in a sustainable way.

> *PERFECT-ER eligible patients new to ward identified. And all beside (sic) folders of these patients checked, and appropriate documentation placed in bedside folders* (SIL Report, Ward D)

The PERFECT-ER checklist was designed to be completed from documentary evidence, however two SILs, again from wards D and E, described using other evidence to complete their checklist.

For peer review only - http://bmjopen.bmj.com/site/about/guidelines.xhtml

…also standing at the end of the bed and looking …so I use quite a lot of things to get the information …you can find it in lots of different places and from obviously talking to staff as well. (SIL, Ward E)

Adaptation

The PERFECT-ER implementation process was adapted in one key area. A ward-level action planning meeting was integral to the PDSA cycle, however none of the SILs held these. A few attempted some meetings:

…in the first sort of four or five months I had meetings with whatever staff I could grab …as time went on umm that got more and more difficult …staff movement …a lot of agency …pool staff (SIL, Ward B)

Ward pressures impacted on staff and their ability to get together as a team as part of implementation. Rather, SILs adapted the process using key, often senior, individuals (such as ward managers or PLs) or people relevant to checklist elements (e.g., physiotherapists for rehabilitation items) to facilitate action planning:

I did manage to get some help from our Orthogeriatrician [PL] *…between the two of us we did a bit of a brainstorm on what we can implement* (SIL, Ward D)

Thus, SILs undertook action planning with ward opinion leaders, but the missing team approach to action planning likely impacted on ward staff opportunities for engagement and reflection, and SIL opportunities to communicate goals and receive feedback on these.

Mechanisms of impact

Multiple mechanisms of impact were identified. A major strategy was peer pressure within the ward environment from SILs who enacted spot checks and audits of whether new or existing intervention practices were carried out. These checks were followed up with reminders, "*nagging*" and/or education at ward level or targeted to the individuals viewed as not complying in comparison to other staff.

Staff are aware that these need updating regularly and these are now part of the weekly ward audits to establish compliance (SIL Report, Ward A)

For peer review only - http://bmjopen.bmj.com/site/about/guidelines.xhtml

...she just nagged us all the time ...she likes to give a hundred percent ...if she thought something were missing she would constantly be reminding you about things (Ward Manager, Ward A)

Spot check on new patient documentation to ensure that items are being used... Remains very hit and miss ...ward staff identified and reminder letter given out (SIL Report, Ward B)

Spent most of the day providing short education sessions to individual staff (SIL Report, Ward C)

These actions suggest that SILs believed in their own self-efficacy in driving and accomplishing implementation goals. Their role provided them the power to assess and if necessary, try to correct ward staffs' implementation actions.

Communication and networking were key to implementation work. SILs created and utilised networks. They both cascaded and acquired information via email correspondence, meetings, presentations to staff members, noticeboard use, education sessions, and ongoing informal communication.

Things that went well: Good networking with other staff who are committed to providing a quality service (SIL Report, Ward C)

Preparing for my presentation for the first of the ward time out days. I have a 45-minute slot to discuss and promote PERFECT-ER (SIL Report, Ward A)

Further discussion required with MDT [Multidisciplinary Team] *to identify who could do this and how, where to record it. Set up a meeting with relevant others* (Action Plan, Ward C)

Ward managers played a key role as gatekeepers of change and SILs waited for their authorisation before implementing any PERFECT-ER elements.

Agreed to meet the senior charge nurse at next visit to get an agreement ...and start the wheels in motion (SIL Report, Ward C)

They and other key clinicians, such as PLs, facilitated implementation using their powerful positions as opinion leaders on the ward to endorse and enforce implementation.

For peer review only - http://bmjopen.bmj.com/site/about/guidelines.xhtml

> *The ward sister* [manager] *has introduced this to the staff and put it in the 'Hot Topics' folder.* (SIL Report, Ward B)

SILs helped staff implement practice changes by making sure new paperwork, assessments and essential equipment were accessible.

> *Ensured adequate supply of new documentation is available in all relevant areas and previous documentation removed from circulation* (SIL Report, Ward C)

Two wards (A and E) enlisted PERFECT-ER "Champions" to take on some responsibility and facilitate implementation while undertaking their usual roles.

> *I then decided to have PERFECT-ER Champions… I can only do so much really …it is like 'right I want you to, you know, it is your responsibility to try and speak to your peers about this'* (SIL, Ward A)

Implementation was enabled by aligning new elements of practice with current practices and organisational initiatives.

> *…putting one of those charts in the Admission Pack …if it is not visible people won't think to use it, so we are trying to put it so as it is normal for them and they will grab a pack and everything is there ready* (SIL, Ward E)

Contextual Factors

The study took place within the context of multiple pressures in all wards. Staffing issues were common, including long-term sickness, high use of agency and bank staff, empty staff positions on wards, and staff being redeployed to other wards. Some wards were affected more than others. Ward A had extra beds on the ward, low staffing levels, and a lack of senior staff due to the ward manager's long-term sickness:

> *This leaves the ward unsafe and staff are struggling to carry out their required duties never mind embrace change and add to their workload* (SIL, Ward A)

This created barriers to implementing PERFECT-ER. Staff changes meant SILs needed to constantly inform new colleagues about PERFECT-ER practices on the ward. A lack of interest from some staff members and resistance to change also reflected points of implementation vulnerability.

For peer review only - http://bmjopen.bmj.com/site/about/guidelines.xhtml

…at times [it] *would demotivate me seeing the lack of engagement from the other staff* (SIL Report, Ward D)

…when the nursing numbers have been so diminished any extra, anything which seems like it might involve extra work is really resisted (PL, Ward A)

I am not sure if that has got off the ground yet there is still a lot of discussion …if people are keen to adopt something there is no discussion it just happens whereas when there is a bit of resistance the discussion goes on and on (Orthogeriatric Doctor, Ward C)

These quotes reflect the relative low priority of PERFECT-ER amongst staff. To compensate, SILs found compatible organisation-level systems and initiatives useful to link PERFECT-ER practices to:

Because it is now part of Trust (the organisation) *documentation it gets done* (SIL, Ward E)

However, organisation-level changes could be slow to materialise leaving ward level changes difficult to enact in isolation.

…it takes time for things like documentation groups to approve these things, Trust-wide approaches and they don't want individual approaches on Wards (PL, Ward E)

DISCUSSION

PERFECT-ER was developed with the aim of enhancing recovery for people with hip fracture and cognitive impairment by making multiple marginal gains in the quality and consistency of care[20-22]. This process evaluation shows that under certain conditions multiple changes can be implemented. Implementing PERFECT-ER appeared to encourage standardised practices and potentially improved consistency of care for this patient group. Key enablers for implementation were found to be: having dedicated person/s with protected time to drive implementation forward; tapping into existing practices; gaining support from key influencers on the ward, utilising networks and in-ward peer pressure. However, implementation vulnerability occurred when ward pressures were considerable, SILs had limited time and ward staff support was lacking, with marginal gains in care delivery appearing unattainable under these conditions. Overcoming barriers in gaining dedicated SIL time may be a prime way to enhance future consistency in implementation.

For peer review only - http://bmjopen.bmj.com/site/about/guidelines.xhtml

Where ward pressures were high and resource for SILs low, dose suffered. The process evaluation findings suggests that although the intervention was standardised, implementation fluctuated across wards and over time and therefore was not optimal. Consistent with other observations, findings indicate the importance of ward context in implementation[28-30,42]. Ward pressures and inadequate SIL resource often contributed to difficulties in implementation. In this study, ward pressures led to adaptations to the implementation process and sometimes to lack of fidelity in implementation. In response to ward pressures all SILs adapted the implementation process by not conducting ward-level action planning meetings as part of the PDSA cycles. Instead, SILs targeted key influencers and opinion leaders, utilising power structures on the wards as mechanisms of impact which may have been stronger to facilitate changes since this approach enabled a successful and streamlined implementation process. Ward staff engagement can be variable even if intervention processes are standardised [43]. This adaptation is likely to have created further losses in ward team engagement, staff reflective practices and commitment and explains why reach was limited in some wards with staff desiring greater involvement with PERFECT-ER implementation. Moving forward, mechanisms for planned staff engagement work should be considered carefully in relation to how and who to engage.

Ongoing interprofessional staff communication in hospitals can take time[44] and relies on individual staff members and supportive organisational structures[30]. In our data, communication work reflected SIL perceptions of self-efficacy through networking with key staff to drive implementation and their employment of peer pressure mechanisms within the ward setting working to obtain compliance. Peer pressure is a component of the CFIR[41], but in relation to competitiveness with other organisations outside of the inner-ward setting. Our data showed a different type of peer pressure, enacted to maximise compliance within the ward setting using power mechanisms provided by the status of the SIL role itself.

Strengths and Limitations

Our study benefits from multiple sources of data collected from a variety of stakeholders to examine implementation. Data encompassed several cycles of implementation, obtaining a comprehensive view of the changes over time, processes of implementation, network usage, and the role of contextual factors in implementation. Considering the challenges involved when caring for patients with hip fracture and cognitive impairment and the consequent

For peer review only - http://bmjopen.bmj.com/site/about/guidelines.xhtml

variability in the quality of care[2,4-7,10-12], our finding that implementing an enhanced recovery checklist appears to encourage standardised practices for this population is promising. Although the findings examined here only relate to five implementation wards, key themes were demonstrated across these and provide a useful starting point for implementation of future complex interventions in acute ward settings and further use of PERFECT-ER.

Limitations to this study include its reliance on SIL-generated data and most patients and carers being unable to compare usual and 'enhanced' care. Patient recruitment for interviews was difficult due to levels of cognitive impairment. Additionally, staff members who participated in interviews or focus groups may have been those more open to the intervention and to change. The PERFECT-ER intervention required SILs to complete the checklist from individual patient documentary evidence, to which researchers did not have access, to provide validation or incorporate assessments of interrater reliability. There may have also been inconsistency in intervention scoring. Future training for SILs to conduct checklist scoring could include assessing interrater reliability, particularly in situations where more than one SIL is appointed. Although participants reported improved standardisation of practices, we cannot ascertain if the indicated implementation reflected change in care delivery, documentation practices or, in some cases, SIL self-implementation. Lastly, this evaluation was of a feasibility, not efficacy trial[20,23], and it is not yet known whether the changes influence patient outcomes.

CONCLUSION

Establishing the conditions under which PERFECT-ER was implemented has made visible potential sources of implementation vulnerability such as staffs' perceived priority of the intervention being low, ward pressures and limited improvement lead resources. Our study suggests that due to pressures, most ward staff were not optimally engaged in implementation processes. Rather key senior staff were enlisted as opinion influencers and improvement leads employed peer pressure to further implementation. Accordingly, existing and new power structures within the ward were used to facilitate implementation. That key individuals successfully endorsed and enforced implementation indicates that whole-ward interventions may not always require cognitive engagement from all ward staff to implement

For peer review only - http://bmjopen.bmj.com/site/about/guidelines.xhtml

changes. Future ward-level implementation studies could consider how best to engage staff and which staff groups to target.

DECLARATIONS

Ethical Approval: Ethical approval for the trial was given by Camden and Kings Research Ethics Committee (reference number: 16/LO/0621) and Scotland Research Ethics Committee A (reference number: 16/SS/0086).

Trial registration number: ISRCTN 99336264.

Funding: This work was supported by NIHR Programme Grants for Applied Research (PGfAR) Programme grant number [ref: DTC-RP-PG-0311-12004].

Contributorship statement

JLC, CF, FP, BP, SPH, and TB made substantial contributions to the conception or design of the work;

SPH, TB and VM-T led on the acquisition of the data;

TB, SPH and JLC led the analysis and interpretation of data for the work;

TB and JLC led the drafting of the paper.

All authors reviewed the paper and gave their final approval of the version to be published;

·All authors give their agreement to be accountable for all aspects of the work in ensuring that questions related to the accuracy or integrity of any part of the work are appropriately investigated and resolved.

There are no competing interests for any author

Data sharing statement:

For peer review only - http://bmjopen.bmj.com/site/about/guidelines.xhtml

Data are available on reasonable request to the corresponding author

REFERENCES

1 World Health Organisation (WHO) *Dementia: a public health priority,* Report: UK 2012. Available from: http://apps.who.int/iris/bitstream/10665/75263/1/9789241564458_eng.pdf?ua=1 (accessed 28 Sep 2018)

2 Gladman J, Porock D, Griffiths A, et al. *Better Mental Health: Care for Older People with Cognitive Impairment in General Hospitals.* Final Report, NIHR Service Delivery and Organisation Programme 2012.

3 Alzheimer's Society. (2009) *Counting the cost*. London: Alzheimer's Society. Available from: https://www.alzheimers.org.uk/sites/default/files/2018-05/Counting_the_cost_report.pdf (accessed 04 Mar 2022).

4 Krupić F, Sadić S, Seffo N, et al. Experience of registered nurses in assessing postoperative pain in hip fracture patients with dementia. *Med Glas (Zenica)* 2018;15:75-80.

5 O'Keeffe S, Lavan J. The Prognostic Significance of Delirium in Older Hospital Patients. *J Am Geriatr Soc* 2015;45(2):174-178.

6 Smith T, Hameed Y, Cross J, *et al.* Assessment of people with cognitive impairment and hip fracture: a systematic review and meta-analysis. *Arch gerontol geriat* 2013;57(2):117-26.

7 Lee HB, Mears SC, Rosenberg PB, *et al.* Predisposing Factors for Postoperative Delirium After Hip Fracture Repair in Individuals with and without Dementia. *J Am Geriatr Soc* 2011;59(12):2306-2313.

8 Seitz DP, Gill SS, Gruneir A, *et al.* Effects of dementia on postoperative outcomes of older adults with hip fractures: a population-based study. *J Am Med Dir Assoc* 2014;15(5):334-41.

9 National Institute for Health and Care Excellence (NICE). *Hip fracture: management, clinical guideline* [CG124] 2011 updated: 2017 https://www.nice.org.uk/guidance/cg124 (accessed 7 March 2022).

For peer review only - http://bmjopen.bmj.com/site/about/guidelines.xhtml

10 Hall AJ, Lang IA, Endacott R. Physiotherapy interventions for people with dementia and a hip fracture—a scoping review of the literature. *Physiotherapy* 2017;103:361-368.

11 Smith T, Yasir AH, Henderson C, et al. Enhanced rehabilitation and care models for adults with dementia following hip fracture surgery (Review). Cochrane Database Syst Rev 2015;6:1-47.

12 Hall AJ, Watkins R, Lang IA, et al. The experiences of physiotherapists treating people with dementia who fracture their hip *BMC Geriatrics* 2017;17:91.

13 Berry SD, Rothbaum RR, Kiel DP, et al. Association of Clinical Outcomes With Surgical Repair of Hip Fracture vs Nonsurgical Management in Nursing Home Residents With Advanced Dementia. *JAMA Intern Med* 2018;178:774–780.

14 Butcher L. Caring for patients with dementia in the acute care setting. *Br J Nurs* 2018;27:358-362.

15 Fogg C, Griffiths P, Meredith P. *et al.* Hospital outcomes of older people with cognitive impairment: An integrative review. *Int J Geriatr Psych*Published Online First: 26 Jun 2018. doi: 10.1002/gps.4919

16 Sampson E, Blanchard M, Jones L, *et al.* Dementia in the acute hospital: Prospective cohort study of prevalence and mortality. *Brit J Psychiat* 2009;195(1):61-66.

17 Gruneir A, Silver MJ, Rochon PA. Emergency department use by older adults: a literature review on trends, appropriateness, and consequences of unmet health care needs. *Med Care Res Rev* 2010;68(2):131-55.

18 Givens JL, Sanft TB, Marcantonio ER. Functional Recovery After Hip Fracture: The Combined Effects of Depressive Symptoms, Cognitive Impairment, and Delirium. *J Am Geriatr Soc* 2008;56:1075-1079.

19 Smith SM, Bayliss EA, Mercer SW, et al. How to design and evaluate interventions to improve outcomes for patients with multimorbidity. *J Comorb* 2013;3:10-17.

20 Hammond SP, Cross J, Shepstone L, et al. Study protocol: Enhancing recovery of patients admitted to acute settings with hip fracture who are identified as experiencing confusion: a multi-centre, cluster-randomised controlled, feasibility trial of the PERFECTED Enhanced Recovery (PERFECT-ER) care versus standard acute care. *Trials* 2017;18:583.

For peer review only

For peer review only - http://bmjopen.bmj.com/site/about/guidelines.xhtml

21 Richards DA. Complex interventions and the amalgamation of marginal gains: A way forward for understanding and researching essential nursing care? *Int J Nurs Stud* 2015;52(7):1143–1145.

22 Durrand JW, Batterham AM, Danjoux GR. Pre-habilitation (i): aggregation of marginal gains. *Anaesthesia* 2014;69:399–419.

23 Cross JL, Hammond SP, Shepstone L, et al. PERFECTED enhanced recovery pathway (PERFECT-ER) versus standard acute hospital care for people after hip fracture surgery who have cognitive impairment: a feasibility cluster randomised controlled trial. *BMJ Open* 2022;12:e055267.

24 Taylor MJ, McNicholas C, Nicolay C, et al. Systematic review of the application of the plan-do-study-act method to improve quality in healthcare. *BMJ Qual Saf* 2014;23:290-98.

25 Speroff T, O'Conner GT. Study Designs for PDSA Quality Improvement Research. *Qual Manag Health Ca* 2004;13(1):17–32.

26 Haynes AB, Weiser TG, Berry WR, et al. Changes in safety attitude and relationship to decreased postoperative morbidity and mortality following implementation of a checklist-based surgical safety intervention. *BMJ Qual Saf* 2011;20:102-107.

27 Lingard L, Espin S, Rubin B, et al. Getting teams to talk: development and pilot implementation of a checklist to promote interprofessional communication in the OR. *BMJ Qual Saf* 2005;14:340-346.

28 Papoutsi C, Poots A, Clements J, et al. Improving patient safety for older people in acute admissions: implementation of the Frailsafe checklist in 12 hospitals across the UK. *Age Ageing* 2018;47:311-317.

29 Bosk CL, Dixon-Woods M, Goeschel CA, et al. Reality check for checklists. *Lancet* 2009;394:444-5.

30 Bridges J, May C, Fuller A, et al. Optimising impact and sustainability: a qualitative process evaluation of a complex intervention targeted at compassionate care. *BMJ Qual Saf* 2017;26:970-977.

31 Moore GF, Audrey S, Barker M, et al. Process evaluation of complex interventions: Medical Research Council guidance. *BMJ* 2015;350:h1258.

For peer review only - http://bmjopen.bmj.com/site/about/guidelines.xhtml

32 Moore GF, Audrey S, Barker M, et al. *Process evaluation of complex interventions: Medical Research Council guidance.* London: MRC Population Health science Research Network 2014.

33 Langley A, Smallman C, Tsoukas H, et al. Process studies of change in organization and management: unveiling temporality, activity, and flow. *Acad Manage J* 2013;56(1):1-13.

34 Cheng KKF. Metcalfe A. Qualitative Methods and Process Evaluation in Clinical Trials Context: Where to Head to? *IJQM* 2018;17:1-4.

35 Murtagh MJ, Thomson RG, May CR, et al. Qualitative methods in a randomised controlled trial: the role of an integrated qualitative process evaluation in providing evidence to discontinue the intervention in one arm of a trial of a decision support tool. *Qual Saf Health Care* 2007;16:224-229.

36 Sutton E, Herbert G, Burden S, et al. Using the Normalization Process Theory to qualitatively explore sense-making in implementation of the Enhanced Recovery After Surgery programme: "It's not rocket science". *PLoS ONE* 2018;13(4):1-14.

37 Campbell NC, Murray E, Darbyshire J, et al. Designing and evaluating complex interventions to improve health care. *BMJ* 2007;334:455.

38 Campbell M, Fitzpatrick R, Haines A, et al. Framework for design and evaluation of complex interventions to improve health. *BMJ* 2000;321:694-696.

39 Skivington K, Matthews L, Simpson SA, et al. Framework for the development and evaluation of complex interventions: gap analysis, workshop and consultation-informed update. *Health Technol Assess* 2021;25(57):1-132.

40 Pinnock H, Barwick M, Carpenter C, et al. Standards for Reporting Implementation Studies (StaRI) statement. *BMJ* 2017;356:i6795

41 Damschroder LJ, Aron DC, Keith RE, et al. Fostering implementation of health services research findings into practice: a consolidated framework for advancing implementation science. *Implementat Sci* 2009;4(50):1-15.

42 Szymczak JE. Beyond barriers and facilitators: the central role of practical knowledge and informal network in implementing infection prevention interventions *BMJ Qual Saf* 2018;27:763-765.

For peer review only - http://bmjopen.bmj.com/site/about/guidelines.xhtml

43 Sheard L, Marsh C, O'Hara J, et al. Exploring how ward staff engage with the implementation of a patient safety intervention: a UK-based qualitative process evaluation. *BMJ Open* 2017;7:e014558. doi: 10.1136/bmjopen-2016-014558

44 Kummerow Broman K, Kensinger C, Hart H. et al. Closing the loop: a process evaluation of inpatient care team communication. *BMJ Qual Saf* 2015;26:30-32.

Figure and Table Legends

Figure 1: Process Evaluation Data Collection

Table 1: Participant Demographics

Figure 2: PERFECT-ER checklist scores by percentage and implementation site

Supplementary table 1

For peer review only

For peer review only - http://bmjopen.bmj.com/site/about/guidelines.xhtml

	PERFECT-ER checklist scores *n*	SIL action plans *n*	SIL reflective reports *n*	Patient interviews *n*	Relative interviews *n*	Ward staff participants (interviews/ focus groups) *n*
**Ward A**	8	7	21	2	8	10
**Ward B**	8	7	21	0	5	13
**Ward C**	8	6	20	1	5	12
**Ward D**	8	6	15	0	4	15
**Ward E**	8	5	15	0	7	13
**Total**	**40**	**31**	**92**	**3**	**29**	**63**

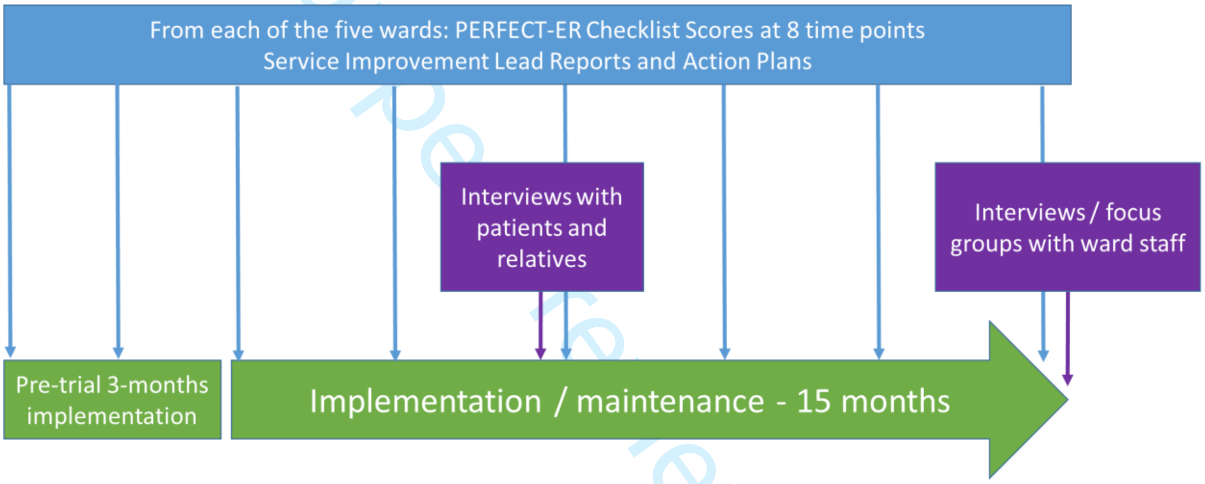

Figure 1: Process Evaluation Data Collection

For peer review only

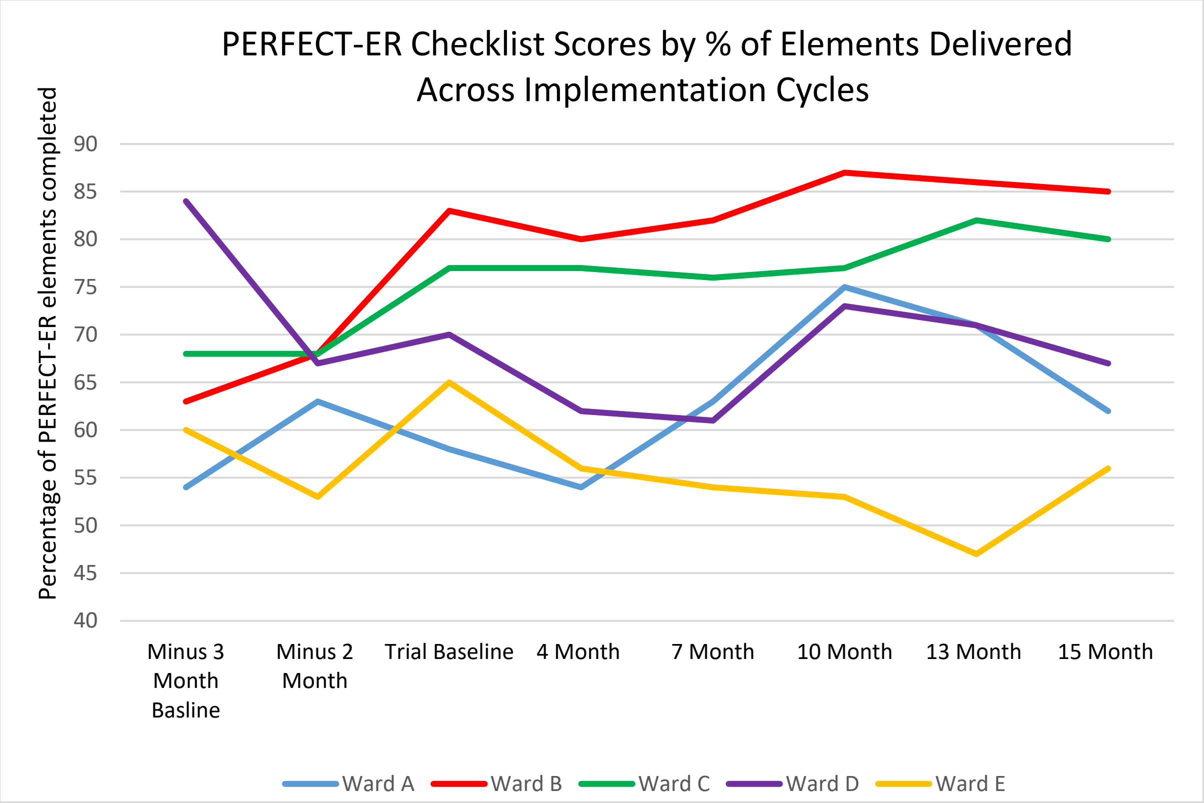

Figure 2: PERFECT-ER checklist scores by percentage and implementation site

For peer review only

Standards for Reporting Implementation Studies: the StaRI checklist for completion

The StaRI standard should be referenced as: Pinnock H, Barwick M, Carpenter C, Eldridge S, Grandes G, Griffiths CJ, Rycroft-Malone J, Meissner P, Murray E, Patel A, Sheikh A, Taylor SJC for the StaRI Group. Standards for Reporting Implementation Studies (StaRI) statement. *BMJ* 2017;356:i6795

The detailed Explanation and Elaboration document, which provides the rationale and exemplar text for all these items is: Pinnock H, Barwick M, Carpenter C, Eldridge S, Grandes G, Griffiths C, Rycroft-Malone J, Meissner P, Murray E, Patel A, Sheikh A, Taylor S, for the StaRI group. Standards for Reporting Implementation Studies (StaRI). Explanation and Elaboration document. *BMJ Open* 2017 2017;7:e013318

Notes: A key concept of the StaRI standards is the dual strands of describing, on the one hand, the implementation strategy and, on the other, the clinical, healthcare, or public health intervention that is being implemented. These strands are represented as two columns in the checklist.

The primary focus of implementation science is the implementation strategy (column 1) and the expectation is that this will always be completed.	The evidence about the impact of the intervention on the targeted population should always be considered (column 2) and either health outcomes reported or robust evidence cited to support a known beneficial effect of the intervention on the health of individuals or populations.

The StaRI standardsrefers to the broad range of study designs employed in implementation science. Authors should refer to other reporting standards for advice on reporting specific methodological features. Conversely, whilst all items are worthy of consideration, not all items will be applicable to, or feasible within every study.

Checklist item		Reported on page #	Implementation Strategy	Reported on page #	Intervention
			"Implementation strategy" refers to how the intervention was implemented		"Intervention" refers to the healthcare or public health intervention that is being implemented.
**Title and abstract**					
Title	**1**	1	Identification as an implementation study, and description of the methodology in the title and/or keywords		
Abstract	**2**	2	Identification as an implementation study, including a description of the implementation strategy to be tested, the evidence-based intervention being implemented, and defining the key implementation and health outcomes.		
**Introduction**					
Introduction	**3**	4	Description of the problem, challenge or deficiency in healthcare or public health that the intervention being implemented aims to address.		
Rationale	**4**	4/5	The scientific background and rationale for the implementation strategy (including any underpinning theory/framework/model, how it is expected to achieve its effects and any pilot work).	5	The scientific background and rationale for the intervention being implemented (including evidence about its effectiveness and how it is expected to achieve its effects).

For peer review only - http://bmjopen.bmj.com/site/about/guidelines.xhtml

Aims and objectives	**5**	5	The aims of the study, differentiating between implementation objectives and any intervention objectives.		
**Methods: description**					
Design	**6**	6-8	The design and key features of the evaluation, (cross referencing to any appropriate methodology reporting standards) and any changes to study protocol, with reasons		
Context	**7**	4-6	The context in which the intervention was implemented. (Consider social, economic, policy, healthcare, organisational barriers and facilitators that might influence implementation elsewhere).		
Targeted 'sites'	**8**	6	The characteristics of the targeted 'site(s)' (e.g locations/personnel/resources etc.) for implementation and any eligibility criteria.	4	The population targeted by the intervention and any eligibility criteria.
Description	**9**	5	A description of the implementation strategy	4-5	A description of the intervention
Sub-groups	**10**	NA	Any sub-groups recruited for additional research tasks, and/or nested studies are described		
**Methods: evaluation**					
Outcomes	**11**	NA Feasibility	Defined pre-specified primary and other outcome(s) of the implementation strategy, and how they were assessed. Document any pre-determined targets	NA Trial paper	Defined pre-specified primary and other outcome(s) of the intervention (if assessed), and how they were assessed. Document any pre-determined targets
Process evaluation	**12**	5	Process evaluation objectives and outcomes related to the mechanism by which the strategy is expected to work		
Economic evaluation	**13**	NA Trial paper	Methods for resource use, costs, economic outcomes and analysis for the implementation strategy	NA Trial paper	Methods for resource use, costs, economic outcomes and analysis for the intervention
Sample size	**14**	7	Rationale for sample sizes (including sample size calculations, budgetary constraints, practical considerations, data saturation, as appropriate)		
Analysis	**15**	8	Methods of analysis (with reasons for that choice)		
Sub-group analyses	**16**	8	Any a priori sub-group analyses (e.g. between different sites in a multicentre study, different clinical or demographic populations), and sub-groups recruited to specific nested research tasks		

For peer review only - http://bmjopen.bmj.com/site/about/guidelines.xhtml

**Results**					
Characteristics	**17**	8-9	Proportion recruited and characteristics of the recipient population for the implementation strategy	Na Trial paper	Proportion recruited and characteristics (if appropriate) of the recipient population for the intervention
Outcomes	**18**	10	Primary and other outcome(s) of the implementation strategy	NA Trial Paper	Primary and other outcome(s) of the Intervention (if assessed)
Process outcomes	**19**	10-16	Process data related to the implementation strategy mapped to the mechanism by which the strategy is expected to work		
Economic evaluation	**20**	NA Trial paper	Resource use, costs, economic outcomes and analysis for the implementation strategy	NA Trial paper	Resource use, costs, economic outcomes and analysis for the intervention
Sub-group analyses	**21**	10	Representativeness and outcomes of subgroups including those recruited to specific research tasks		
Fidelity/ adaptation	**22**	11-13	Fidelity to implementation strategy as planned and adaptation to suit context and preferences	10	Fidelity to delivering the core components of intervention (where measured)
Contextual changes	**23**	13-14	Contextual changes (if any) which may have affected outcomes		
Harms	**24**	NA Trial paper	All important harms or unintended effects in each group		
**Discussion**					
Structured discussion	**25**	16-17	Summary of findings, strengths and limitations, comparisons with other studies, conclusions and implications		
Implications	**26**	16-18	Discussion of policy, practice and/or research implications of the implementation strategy (specifically including scalability)		Discussion of policy, practice and/or research implications of the intervention (specifically including sustainability)
**General**					
Statements	**27**	3	Include statement(s) on regulatory approvals (including, as appropriate, ethical approval, confidential use of routine data, governance approval), trial/study registration (availability of protocol), funding and conflicts of interest		

For peer review only

Supplementary Figure 1: PERFECT-ER logic model

Research problem: People with hip fracture and cognitive impairment have poorer outcomes than those without cognitive impairment.

Process outputs: Changes to Ward Practices; Changes to Ward Ethos

Efficacy outputs: Staff Impacts & Outcomes; Patient Impacts & Outcomes; Carer Impacts & Outcomes

PERFECT-ER Inputs

- SIL .5 for 3 months, then .2 for duration
- SIL 3 day induction training
- PPL .01 per week for 3 months, then .01 per month
- PERFECT-ER Checklist
- PERFECT-ER Manual
- University Staff Member to contact
- Administration / equipment costs

PERFECT-ER Processes and Activities

- SIL completes PERFECT-ER Checklist
- SIL collates scores (Figure 2)
- Ward staff action planning
- SIL works with ward staff to implement PERFECT-ER elements
- Repeat
- SIL raises profile of PERFECT-ER on ward

Changes to Ward Practices

- Adapted practices
- Ward staff using new procedures
- Ward staff documenting actions more
- Standardised practices
- Multiple increased aspects of care such as, carer involvement, more rehabilitation etc

Changes to Ward Ethos

- Embedded re-enablement culture
- Increased staff awareness of the needs of patients with cognitive impairments
- Staff empowerment to make changes and improve care
- Cognitive impairment friendly ward

Staff Impacts & Outcomes

- Staff adapt their practices
- Increased reflective practice
- More / changed work practices
- Staff feel empowered to make changes
- Staff feel part of a stronger team
- Staff demonstrate competence and confidence when looking after cognitively impaired patients

Patient Impacts & Outcomes

- Patients receive better care
- Patients feel better looked after
- Patients have access to more aspects of care
- Patients have better clinical (including 30 day survival) and cognitive outcomes
- Patients have improved quality of life

Carer Impacts & Outcomes

- Carers feel included in and consulted about patient care
- Carers have reduced anxiety about patient care
- Carers know what is happening and what to expect in relation to patient care

Key
SIL: Service Improvement Lead (Generally a qualified staff member)
PL: Process Lead (Consultant level)

Assumptions: Embedding and sustaining numerous small improvements to hip fracture service delivery optimised for patients with CI will standardise care delivery, improving staff confidence and care delivery, leading to improved patient outcomes and patient and carer experience.

External factors that may influence implementation:

- Chronic bed shortages meaning patients on outlier wards (e.g. Winter pressures)
- Chronic staff shortages
- Local organisation policies
- Changes to national guidance

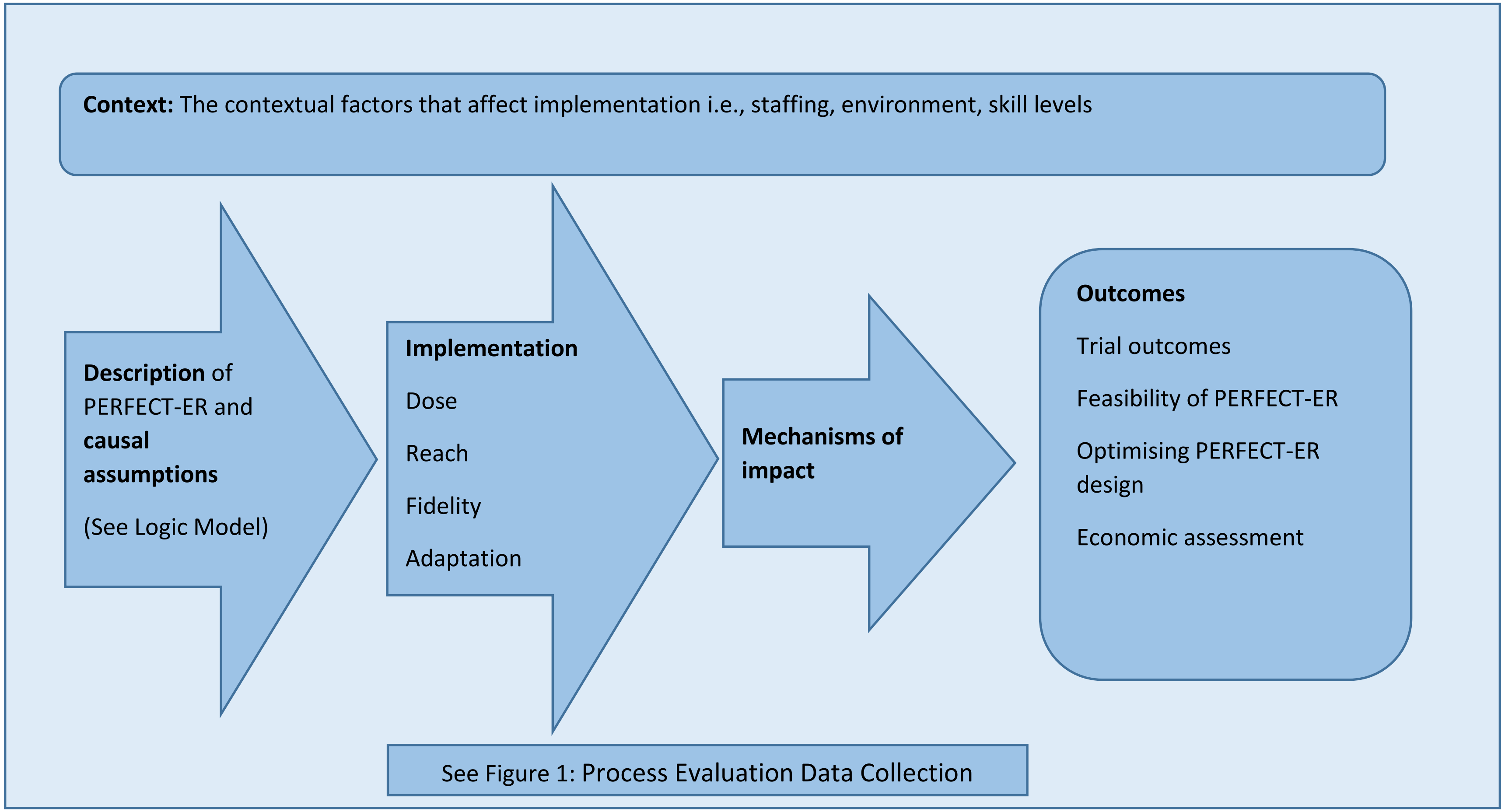
Context: The contextual factors that affect implementation i.e., staffing, environment, skill levels
Description of PERFECT-ER and **causal assumptions**
(See Logic Model)
Implementation
Dose
Reach
Fidelity
Adaptation
Mechanisms of impact
Outcomes
Trial outcomes
Feasibility of PERFECT-ER
Optimising PERFECT-ER design
Economic assessment
See Figure 1: Process Evaluation Data Collection

BMJ Open

Implementing an intervention to enhance care delivery and consistency for people with hip fracture and cognitive impairment in acute hospital wards: a mixed methods process evaluation of a randomised controlled feasibility trial (PERFECTED)

Journal:	*BMJ Open*
Manuscript ID	bmjopen-2022-064482.R1
Article Type:	Original research
Date Submitted by the Author:	20-Dec-2022

Complete List of Authors:	Backhouse, Tamara; University of East Anglia, School of Health Sciences<br>Fox, Chris; Norwich Medical School, Department of Psychological Sciences; University of Exeter,<br>Hammond, Simon; University of East Anglia, Norwich Medical School<br>Poland, Fiona; University of East Anglia, School of Health Sciences<br>McDermott-Thompson, Vicki; University of East Anglia<br>Penhale, Bridget; University of East Anglia<br>Cross, Jane; University of East Anglia

**Primary Subject Heading**:	Health services research
Secondary Subject Heading:	Geriatric medicine, Patient-centred medicine, Qualitative research
Keywords:	Dementia < NEUROLOGY, Delirium & cognitive disorders < PSYCHIATRY, ORTHOPAEDIC & TRAUMA SURGERY

SCHOLARONE™ Manuscripts

I, the Submitting Author has the right to grant and does grant on behalf of all authors of the Work (as defined in the below author licence), an exclusive licence and/or a non-exclusive licence for contributions from authors who are: i) UK Crown employees; ii) where BMJ has agreed a CC-BY licence shall apply, and/or iii) in accordance with the terms applicable for US Federal Government officers or employees acting as part of their official duties; on a worldwide, perpetual, irrevocable, royalty-free basis to BMJ Publishing Group Ltd ("BMJ") its licensees and where the relevant Journal is co-owned by BMJ to the co-owners of the Journal, to publish the Work in this journal and any other BMJ products and to exploit all rights, as set out in our licence.

The Submitting Author accepts and understands that any supply made under these terms is made by BMJ to the Submitting Author unless you are acting as an employee on behalf of your employer or a postgraduate student of an affiliated institution which is paying any applicable article publishing charge ("APC") for Open Access articles. Where the Submitting Author wishes to make the Work available on an Open Access basis (and intends to pay the relevant APC), the terms of reuse of such Open Access shall be governed by a Creative Commons licence – details of these licences and which Creative Commons licence will apply to this Work are set out in our licence referred to above.

Other than as permitted in any relevant BMJ Author's Self Archiving Policies, I confirm this Work has not been accepted for publication elsewhere, is not being considered for publication elsewhere and does not duplicate material already published. I confirm all authors consent to publication of this Work and authorise the granting of this licence.

For peer review only

BMJ OPEN TITLE PAGE

Title: Implementing an intervention to enhance care delivery and consistency for people with hip fracture and cognitive impairment in acute hospital wards: a mixed methods process evaluation of a randomised controlled feasibility trial (PERFECTED)

Authors and Affiliations:

Tamara Backhouse[1] Chris Fox[2] Simon P Hammond[3] Fiona Poland[1] Vicki McDermott-Thompson[4] Bridget Penhale[1] Jane L Cross[1]*

1 School of Health Sciences, University of East Anglia, Norwich Research Park, Norwich, UK

2 College of Medicine and Health, University of Exeter, Exeter, EX1 2HZ, UK

3 School of Education & Lifelong Learning, University of East Anglia, Norwich Research Park, Norwich, UK

4 School of Psychology, University of East Anglia, Norwich Research Park, Norwich, UK

***Corresponding author's contact details:**

Dr Jane L. Cross

Email: j.cross@uea.ac.uk

Key words: Cognitive impairment, Dementia, hip fracture, Implementation, acute hospital, Process evaluation

Manuscript 3966 words

Abstract 300 words

Tables 1

Figures 2

Supplementary tables 1

For peer review only

STRUCTURED ABSTRACT:

Objectives: To determine how, and under what circumstances, the PERFECT-ER intervention was implemented in five acute hospital wards and impacted on staff practices and perceptions.

Design: Mixed methods process evaluation (undertaken between 2016-18).

Setting: Five acute hospital wards across three different UK regions.

Participants: Patients (n=3) admitted to acute wards with hip fracture and cognitive impairment, their relatives (n=29) and hospital staff (n=63).

Interventions: PERFECT-ER, a multi-component intervention designed to enhance the recovery of patients with hip fracture and cognitive impairment was implemented for 18-months. PERFECT-ER was implemented at ward level ensuring that multiple new and existing practices were undertaken consistently, on the assumption that collectively, small individual advances would improve care delivery for patients.

Primary and secondary outcome measures: Implementation of the PERFECT-ER intervention examined through regular intervention scores, service improvement staff reports and action plans, and semi-structured interviews and focus groups.

Results: The process evaluation identified points of implementation vulnerability and strength. All wards implemented some elements of PERFECT-ER. Implementation was fragile when ward pressures were high and when ward staff perceived the relative priority of intervention practices to be low. Adaptations to the implementation process may have reduced whole-ward staff engagement with implementation. However, strategic enlistment of senior ward influencers (such as ward managers, orthogeriatricians) combined with service improvement lead in-ward peer pressure tactics facilitated implementation processes.

Conclusions: Our study suggests that implementation was expediated when senior staff were onboard as opinion leaders and formally appointed internal implementation leaders exerted their power. Within hierarchical settings such as acute wards, key individuals appeared to influence implementation through endorsement and sometimes enforcement. This indicates that whole-ward interventions may not always require cognitive engagement from all ward

For peer review only - http://bmjopen.bmj.com/site/about/guidelines.xhtml

staff to implement changes. Future ward-level implementation studies could consider how best to engage staff and most importantly, which staff to best target.

Trial registration: ISRCTN99336264

Strengths and limitations:

- Strengths of this study include obtaining data from multiple sources and stakeholders from five different acute hospital wards across seven implementation cycles.
- Another strength was the independence of the core process evaluation team from the feasibility trial team.
- Due to the nature of the intervention, documentary evidence from patients' notes was used by improvement leads to complete intervention checklists and this information could not be verified by researchers.
- The research team could not confirm whether implementation was due to changes in clinical care, documenting practices or due to improvement leads' own actions.

Funding statement: This work was supported by NIHR Programme Grants for Applied Research (PGfAR) Programme grant number [ref: DTC-RP-PG-0311-12004].

Ethical Approval: Ethical approval for the trial was given by Camden and Kings Research Ethics Committee (reference number: 16/LO/0621) and Scotland Research Ethics Committee A (reference number: 16/SS/0086).

Competing interests statement: The authors declare they have no competing interests.

For peer review only

Background

The increasing incidence of dementia is a world-wide public health challenge, requiring multiple strategies including action to improve the quality of healthcare services[1]. Providing acute care for patients with cognitive impairment (including those with either diagnosed or assumed dementia and/or delirium) is challenging for hospital staff[2,3]. Patients with cognitive impairment have complex needs related to delirium, behaviours that challenge, incontinence, dehydration, and communication difficulties. These make necessary medical, nursing and rehabilitation processes more difficult for staff to undertake[2,4-7].

In acute settings, over 40% of people with a hip fracture have cognitive impairment[8]. Usual biomedical approaches[9] and limited evidence for rehabilitation for these patients[10-12] create further difficulties for staff. Consequently, patients with hip fracture and cognitive impairment have increased risk of unfavourable outcomes[13-16], impaired rehabilitation and recovery[6,17], with a worse prognosis than patients without cognitive impairment[8,18]. Hospital staff need to manage these complexities and complex multifaceted interventions to improve quality in healthcare delivery, and outcomes are increasingly required[19].

The PERFECTED research programme was funded by The National Institute for Health Research (NIHR), UK, to develop and test an intervention to enhance the recovery of patients with hip fracture and cognitive impairment on acute wards. PERFECT-ER is a complex multicomponent service improvement intervention which aims to enhance recovery using multiple marginal gains in the quality of care[20-23]. Marginal gains include the implementation of new practices, standardisation of existing practices, ensuring consistency, and cultivating a whole-ward 'in-it-together' ethos.

The PERFECT-ER intervention comprises a structured checklist [24], Service Improvement Lead (SIL), Process Lead (PL) and implementation manual using a Plan, Do, Study, Act (PDSA) approach to facilitate behaviour change[25-26]see logic model (Supplementary Figure 1). PERFECT-ER is a whole ward approach involving all staff delivering patient care. The PERFECT-ER checklist has 68 practice items (including clinical, functional, psychosocial, and cognitive elements), which span admission, pre-operative, post-operative, rehabilitation, and discharge periods and 15 organisational items, which highlight the policies behind current practices in the hospital. Checklists have been used before to improve the quality of care[27,28], however

For peer review only - http://bmjopen.bmj.com/site/about/guidelines.xhtml

such lists rarely change practice unless combined with mechanisms for change within the socio-practice contexts of hospital settings[29,30]. For example, much relational work in hospitals relies on individual staff members and supportive organisational structures[31].

PERFECT-ER is implemented by a SIL, a formally appointed internal implementation lead, a qualified healthcare professional, based day-to-day on the selected ward. They attend a three-day induction training in intervention delivery. They were supported by a senior doctor acting as a PL to facilitate implementation across the organisation. The checklist, designed to overlay current hip fracture pathways, identifies areas of strength and weaknesses in current practice. It is used to score a sample of patients' notes (documentary evidence) to determine current practice and identify areas for improvement. Scores are shared with staff at action-planning meetings, where discussions create shared action plans to address lower scoring items. The Plan (action plan), Do (implementation), Study (fill in checklist and assess scores), Act (engage ward staff) cycle then recommences.

As part of the wider PERFECTED programme of research, we conducted a process evaluation of the PERFECT-ER feasibility trial[20,23]. The aim of this process evaluation was to determine how and under what circumstances the PERFECT-ER intervention was implemented in acute hospital wards and impacted on staff practices and perceptions (for published protocol, see supplementary material) [20,32-34].

Ethical approval for the PERFECTED feasibility trial and process evaluation was granted by Camden and Kings Research Ethics Committee (reference number: 16/LO/0621) and Scotland Research Ethics Committee A (reference number: 16/SS/0086). The trial was registered with ISRCTN 99336264.

METHODS

Qualitative data from process evaluations of trials are widely used to generate understandings of intervention implementation[35] and can be of critical importance[36]. However, there is limited qualitative evidence about the implementation of complex interventions to enhance recovery in hospital settings[37]. Therefore, to examine implementation in acute wards in detail, we used a mixed methods approach incorporating qualitative data.

For peer review only - http://bmjopen.bmj.com/site/about/guidelines.xhtml

The process evaluation took place in five different acute NHS hospital wards in five separate geographical areas across England and Scotland where the PERFECT-ER intervention was being implemented. Resource use and patient and relative outcomes are reported elsewhere[23]. We used the Medical Research Council guidance[32,33,38-41] for the evaluation and the Standards for Reporting Implementation Studies (StaRI)[42].

Process evaluation data were gathered between August 2016 and March 2018 over seven PDSA cycles (see Figure 1 for data collected from each ward). We collected PERFECT-ER checklist scores, SIL-generated reflective reports of implementation activities and action plans, and semi-structured interview and focus group data from each ward. PERFECT-ER checklist scores were obtained from patient notes by the SILs. At each ward there were eight checklist time-points: 3 months pre-trial, 1.5 months pre-trial, trial baseline, 4 months, 7 months, 10 months, 13 months, and end of trial (15 months).

Figure 1 here

We undertook: semi-structured face-to-face or telephone interviews with hospital staff and relatives of patients; focus groups with hospital staff; and face-to-face interviews with patients. We purposively sampled ward-based staff (up to 15 from each ward) who had been in contact with the intervention. Interviews and focus groups with staff investigated their knowledge of, and engagement with, the intervention and implementation processes. These were undertaken after trial processes finished to prevent interviews impacting on implementation. Patients and relatives were offered the opportunity to take part in interviews by a research nurse during a follow-up visit for the trial. Interviews were designed to explore patients and relatives' experiences of the intervention ward. All had capacity to consent. Patients and relatives' interviews took place in their home or in a private room at the hospital according to their wishes. Interviews and focus groups were conducted by academic researchers not collecting data or consent for the feasibility trial (SPH, TB and VM-T).

Patient and public involvement

For peer review only - http://bmjopen.bmj.com/site/about/guidelines.xhtml

PERFECTED prioritised Public patient involvement (PPI) from grant development to completion. PPI representatives provided feedback on participant information materials and the study protocol, and had roles on the Trial Steering Committee, Data Monitoring Ethics Committee, and Advisory Groups. For the process evaluation, PPI carer representatives co-interviewed, with researchers, in six interviews with relatives. They received training and support to facilitate this.

Analysis

All interviews and focus groups were digitally recorded and transcribed verbatim. Identifying features were removed to ensure confidentiality and anonymity. Qualitative analysis was iterative throughout the data collection and thematic, focussing on implementation processes informed by the MRC Process Evaluations of Complex Interventions[37-39] including dose, reach, fidelity, adaptations, mechanisms of impact, and context and The Consolidated Framework for Implementation Research (CFIR)[43] in regard to aspects of the inner setting (hospital ward), characteristics of individuals, and process. NVivo software was used to manage the data. TB generated initial codes from repeated reading of data, identifying tentative themes, before multiple analysis meetings with the study team (JLC, SPH, FP, VM-T, CF), which refined and developed these analyses both across sites and within individual sites. Quantitative data from PERFECT-ER checklist scores, collected from five patients at each of the five sites at eight timepoints, were analysed descriptively by ward and implementation cycle.

Results

Sixty-nine interviews and 10 focus groups were conducted, Table 1 describes this population.

Table 1: Participant Demographics

Participant	Total n=95 *n*(%)	Female *n*(%)	Age Mean (range)	Method of data collection by *n*		
				Face-to-face interview	Telephone interview	Focus Group
Patient	3(3)	3(100)	78(68-87)	3	0	0
Relatives*	29(31)	22(76)	57(22-75)	7	22	0
Service Improvement Lead	6(6)	5(83)	45(29-59)	1	5	0

For peer review only - http://bmjopen.bmj.com/site/about/guidelines.xhtml

Process Lead (Consultant Geriatricians)	5(5)	1(20)	44 (37-49)	0	5	0
Research Nurse	8(8)	8(100)	43(26-54)	0	6	2
Hospital staff**	44(46)	43(98)	39(22-60)	19	1	24

**daughter (n=15), son (n=3), daughter-in-law (n=4), son-in-law (n=3), niece (n=1), granddaughter (n=1), husband (n=1), sister-in-law (n=1)*

***nurse (n=12), senior nurse (n=9), healthcare assistant (n=8), occupational health therapist (n=4), physiotherapist (n=3), ward administrator (n=2), ward housekeeper (n=2), doctor (n=1), trauma assistant (n=1), general therapy assistant (n=1), clinical trial assistant (n=1)*

Dose

Figure 2 shows PERFECT-ER checklist scores for each ward by percentage of elements delivered throughout the study. All wards implemented some elements of PERFECT-ER. Dose varied across wards. Availability of resources appeared to help or hinder implementation. Wards B and C implemented well (PERFECT-ER scores increasing 22% and 12% respectively over the study). Wards B and C had dedicated SIL time and clear ward processes which could be utilised for implementation. Wards A (PERFECT-ER scores increasing 8%), D (PERFECT-ER scores decreasing 7%) and E (PERFECT-ER scores decreasing 9%) struggled with severe ward pressures, including staff shortages, affecting implementation. Wards D and E also had difficulties providing dedicated SIL time and the implementation climate reflected a lack of consistent support from ward staff. Ward D had a different SIL obtain the baseline data from the rest of the cycles. Average dose delivered was 68%. PERFECT-ER practice items least implemented were: 1) carers given the opportunity to accompany the patient in the recovery room, and 2) pain assessed daily using a pain scale specific to patients with cognitive impairment.

Figure 2 here

Reach

A proportion of ward staff at all sites engaged with PERFECT-ER to some degree. Many staff at Ward C were largely unaware of the study and when asked about changes due to the intervention their typical response was:

> Nurse Practitioner: *I wouldn't have thought, not through PERFECT-ER no, I wouldn't think so no*

For peer review only - http://bmjopen.bmj.com/site/about/guidelines.xhtml

Senior Nurse: *Or if it is through PERFECT-ER no-one has told us it has come from that* (Focus Group, Ward C)

Nevertheless, PERFECT-ER elements implemented for the study were now part of their usual daily practice and valued.

Nurse Practitioner: *…all the tools that we are bringing in are certainly useful to help appropriately nurse the patients that we actually do have* (Focus Group, Ward C)

This suggests that implementation of PERFECT-ER elements did not rely on the engagement of all ward staff with the process.

In wards C and E several staff members said they would have liked more information about the intervention and an active role with implementation.

Physiotherapist: *Just to know more about it* [PERFECT-ER] *really… So, we could get more involved basically.* (Focus Group, Ward E)

This suggests optimal reach was not always achieved. Nevertheless, across wards elements of PERFECT-ER were viewed as having improved staff awareness, particularly of pain and delirium.

Occupational Therapist: *I suppose it* [PERFECT-ER] *has highlighted it* [pain] *individually to me to be more aware and do something about it.* (Focus Group, Ward C)

Interviews with relatives and patients suggested they did not notice an enhanced care experience. They expressed mixed experiences of care generally, but no-one noted PERFECT-ER specifically in their interview.

By and large as I say it was a good experience umm I'd, I had done it before… I think it was better than it was four years ago (Relative, Ward C)

We felt like we were being a negative complaining family in a way because things just weren't going right (Relative, Ward A)

Indicators of implementation may have reflected changes in documentary practices, and not changes in ethos or clinical practice as staff noted modifications to standardise recording and assessment practices.

For peer review only - http://bmjopen.bmj.com/site/about/guidelines.xhtml

I don't think it changed my practice… it helped standardise those that perhaps wouldn't routinely do the same thing, but for those that were doing it, it allowed us to be clearly documented (PL, Ward E)

Fidelity

Changes in ward ethos varied and appeared to be linked to the implementation climate, dose delivered and reach. Staff at ward C, where implementation went well, talked about changes in approach.

Occupational Therapist: *I think as* [senior nurse] *highlighted, it's* [PERFECT-ER] *brought an awareness of delirium… I have had a sense of that amongst the nursing colleagues …people are a lot more sensitive and patient and maybe provide better care maybe.*

Senior Nurse: *Yeah I agree*

(Focus Group, Ward C)

Some participants indicated PERFECT-ER provided a guiding philosophy for staff.

PERFECT-ER is our ward philosophy do you know what I mean? … it actually is part of their [Ward staff's] *umm vision part of their thinking about their patient care* (SIL, Ward A)

Whereas, in Ward E which struggled to implement, there was no ethos change.

Occupational Therapist: *As far as the PERFECTED Ward goes I am not sure how much it has really, it has sort of changed our practice as such because we kind of work to enable people anyway…*

Lead Occupational Therapist: *Yeah, I am still not a hundred percent clear about the different check lists …that makes it the PERFECTED Ward …I don't think it is something that is obvious what we are working towards.*

(Focus Group, Ward E)

Many aspects of PERFECT-ER were implemented as intended. However, the intervention was developed with one SIL intended as the appointed implementation leader. Whereas two wards employed three individuals to conduct the SIL role between them.

For peer review only - http://bmjopen.bmj.com/site/about/guidelines.xhtml

I do not feel I have been very supported by the other SILs …due to workload on the project ward and lack of staff (SIL, Ward E)

…my management team, they tried to give an hour or two on occasional days …subject to how busy the ward was so found that majority of the times this ended up not being used (SIL Report, Ward D)

In these wards, readiness for implementation was limited by organisational commitment of resources. SIL time was not protected and teamwork between SILs was lacking.

Two SILs from these wards (D and E) changed their own practices by ensuring PERFECT-ER documents were present in patients' notes instead of encouraging ward practices to change in a sustainable way.

…folders of these patients checked, and appropriate documentation placed in bedside folders (SIL Report, Ward D)

The PERFECT-ER checklist was designed to be completed from documentary evidence, however two SILs, again from wards D and E, described using other evidence to complete their checklist.

…also standing at the end of the bed and looking …so I use quite a lot of things to get the information …you can find it in lots of different places and from obviously talking to staff as well. (SIL, Ward E)

Adaptation

The PERFECT-ER implementation process was adapted significantly from that tested (ref to wp2 paper) . A ward-level action planning meeting was integral to the PDSA cycle, however none of the SILs held these. A few attempted some meetings.

…in the first sort of four or five months I had meetings with whatever staff I could grab …as time went on umm that got more and more difficult …staff movement …a lot of agency …pool staff (SIL, Ward B)

Ward pressures impacted on staff and their ability to get together as a team as part of implementation. Rather, SILs adapted the process using senior individuals (such as ward

For peer review only - http://bmjopen.bmj.com/site/about/guidelines.xhtml

managers or PLs) or people relevant to checklist elements (e.g., physiotherapists for rehabilitation items) to facilitate action planning.

> *I did manage to get some help from our Orthogeriatrician* [PL] *…between the two of us we did a bit of a brainstorm on what we can implement* (SIL, Ward D)

Thus, SILs undertook action planning with ward opinion leaders, but the missing team approach to action planning probably impacted on ward staff opportunities for engagement.

Mechanisms of impact

Multiple mechanisms of impact were identified. A major strategy was peer pressure within the ward environment from SILs who enacted spot checks and audits of whether new or existing intervention practices were carried out, often followed up with reminders, "*nagging*" and/or education.

> *Staff are aware that these need updating regularly and these are now part of the weekly ward audits to establish compliance* (SIL Report, Ward A)

> *…she just nagged us all the time* (Ward Manager, Ward A)

> *Spot check on new patient documentation to ensure that items are being used… Remains very hit and miss …ward staff identified and reminder letter given out* (SIL Report, Ward B)

> *Spent most of the day providing short education sessions to individual staff* (SIL Report, Ward C)

These actions suggest that SILs, as formally appointed internal implementation leaders, believed in their own self-efficacy in driving and accomplishing implementation goals.

Networks and communication were key to implementation work. SILs created and utilised networks; cascading and acquiring information via email correspondence, meetings, presentations to staff members, noticeboard use, education sessions, and ongoing informal communication.

> *Good networking with other staff who are committed to providing a quality service* (SIL Report, Ward C)

For peer review only - http://bmjopen.bmj.com/site/about/guidelines.xhtml

Preparing for my presentation for the first of the ward time out days. I have a 45-minute slot to discuss and promote PERFECT-ER (SIL Report, Ward A)

Ward managers played a key role as gatekeepers of change and SILs waited for their authorisation before implementing any PERFECT-ER elements.

Agreed to meet the senior charge nurse at next visit to get an agreement …and start the wheels in motion (SIL Report, Ward C)

They and other key clinicians, such as PLs, facilitated implementation using their powerful positions as opinion leaders on the ward to endorse and enforce implementation.

The ward sister [manager] *has introduced this to the staff and put it in the 'Hot Topics' folder.* (SIL Report, Ward B)

Two wards (A and E) enlisted PERFECT-ER "Champions" to take on some responsibility and facilitate implementation while undertaking their usual roles.

I then decided to have PERFECT-ER Champions… I can only do so much really …it is like 'right I want you to, you know, it is your responsibility to try and speak to your peers about this' (SIL, Ward A)

Implementation was enabled by aligning new elements of practice with current practices and organisational initiatives.

…putting one of those charts in the Admission Pack …if it is not visible people won't think to use it, so we are trying to put it so as it is normal for them and they will grab a pack and everything is there ready (SIL, Ward E)

Contextual Factors

The study took place within the context of multiple pressures in all wards. Staffing issues were common. Ward A had extra beds on the ward, low staffing levels, and a lack of senior staff due to the ward manager's long-term sickness:

This leaves the ward unsafe and staff are struggling to carry out their required duties never mind embrace change and add to their workload (SIL, Ward A)

For peer review only - http://bmjopen.bmj.com/site/about/guidelines.xhtml

This created barriers to implementing PERFECT-ER. Staff changes meant SILs needed to constantly engage with and inform new colleagues about PERFECT-ER practices on the ward.

The implementation climate in the inner setting involved a lack of interest from some staff members and resistance to change, which reflected points of implementation vulnerability.

> *…at times* [it] *would demotivate me seeing the lack of engagement from the other staff* (SIL Report, Ward D)

> *…when the nursing numbers have been so diminished any extra, anything which seems like it might involve extra work is really resisted* (PL, Ward A)

These quotes reflect the relative low priority of PERFECT-ER amongst staff.

Similarly, organisation-level changes could be slow to materialise leaving ward-level changes difficult to enact in isolation.

> *…it takes time for things like documentation groups to approve these things, Trust-wide approaches and they don't want individual approaches on Wards* (PL, Ward E)

Lessons learned from this process evaluation to address consistency in intervention implementation and participant retention for a future definitive trial are reported in Figure 3.

Figure 3 here

DISCUSSION

PERFECT-ER was developed with the aim of enhancing recovery for people with hip fracture and cognitive impairment by making multiple marginal gains in the quality and consistency of care[20-22]. This process evaluation shows that under certain conditions multiple changes can be implemented leading to multiple marginal gains in quality and consistency and changes in ward ethos. Implementing PERFECT-ER appeared to encourage standardised practices and potentially improved consistency of care for this patient group. Key enablers for implementation were found to be: having dedicated person/s with protected time to drive implementation forward; tapping into existing practices; gaining support from opinion leaders on the ward, utilising networks and in-ward peer pressure. However, implementation vulnerability occurred when ward pressures were considerable, SILs had limited time and ward staff support was lacking, with marginal gains in care delivery appearing unattainable

For peer review only - http://bmjopen.bmj.com/site/about/guidelines.xhtml

under these conditions. Overcoming barriers in gaining dedicated SIL time may be a prime way to enhance future consistency in implementation. Staff availability has been identified as a factor in other process evaluations related to hip fracture patients[43, 44].

Where ward pressures were high and resource for SILs low, dose suffered. The process evaluation findings suggests that although the intervention was standardised, implementation fluctuated across wards and over time and therefore was not optimal. Consistent with other observations, findings indicate the importance of ward context in implementation[29-31,45]. Ward pressures and inadequate SIL resource contributed to adaptations to the implementation process and sometimes to lack of fidelity in implementation. In response to ward pressures all SILs adapted the implementation process by not conducting ward-level action planning meetings as part of the PDSA cycles. Instead, SILs targeted opinion leaders, utilising power structures on the wards as mechanisms of impact which may have been stronger to facilitate changes since this approach enabled a successful and streamlined implementation process. Ward staff engagement can be variable even if intervention processes are standardised [46]. This adaptation is likely to have created further losses in ward team engagement, staff reflective practices and commitment and explains why reach was limited in some wards with staff desiring greater involvement with PERFECT-ER implementation. Moving forward, mechanisms for planned staff engagement work should be considered carefully in relation to how and who to engage.

Ongoing interprofessional staff communication in hospitals can take time[47] and relies on individual staff members and supportive organisational structures[31]. In our data, communication work reflected SIL perceptions of self-efficacy through networking with key staff to drive implementation and their employment of peer pressure mechanisms within the ward setting working to obtain compliance. Peer pressure is a component of the CFIR[42], but in relation to competitiveness with other organisations outside of the inner-ward setting. Our data showed a different type of peer pressure, enacted to maximise compliance within the ward setting using power mechanisms provided by the status of the SIL role itself.

Strengths and Limitations

Our study benefits from multiple sources of data collected from a variety of stakeholders to examine implementation. Data collection involved PPI acting as co-interviewers. Data

For peer review only - http://bmjopen.bmj.com/site/about/guidelines.xhtml

encompassed several cycles of implementation, obtaining a comprehensive view of the changes over time, processes of implementation, network usage, and the role of contextual factors in implementation. Considering the challenges involved when caring for patients with hip fracture and cognitive impairment and the consequent variability in the quality of care[2,4-7,10-12], our finding that implementing an enhanced recovery checklist appears to encourage standardised practices for this population is promising. Although the findings examined here only relate to five implementation wards, key themes were demonstrated across these and provide a useful starting point for implementation of future complex interventions in acute ward settings and further use of PERFECT-ER.

Limitations to this study include its reliance on SIL-generated data and most patients and carers being unable to compare usual and 'enhanced' care. Patient recruitment for interviews was difficult due to levels of cognitive impairment, resulting in few patient participants. Most relatives interviewed were one generation younger than the patients, consequently their opinions regarding hospital care may differ. Additionally, staff members who participated in interviews or focus groups may have been those more open to the intervention and to change. The PERFECT-ER intervention required SILs to complete the checklist from individual patient documentary evidence to which researchers did not have access, to provide validation or incorporate assessments of interrater reliability. There may have also been inconsistency in intervention scoring. Although participants reported improved standardisation of practices, we cannot ascertain if the indicated implementation reflected change in care delivery, documentation practices or, in some cases, SIL self-implementation. Lastly, this evaluation was of a feasibility, not efficacy trial[20,23], and it is not yet known whether the changes influence patient outcomes. This may have reduced the motivation of hospital ward staff to implement PERFECT-ER.

CONCLUSION

Establishing the conditions under which PERFECT-ER was implemented has made visible potential sources of implementation vulnerability such as staffs' perceived priority of the intervention being low, ward pressures and limited improvement lead resources. Our study suggests that due to pressures, most ward staff were not optimally engaged in implementation processes. Rather key senior staff were enlisted as opinion leaders and improvement leads employed peer pressure to further implementation. That key individuals

For peer review only - http://bmjopen.bmj.com/site/about/guidelines.xhtml

successfully endorsed and enforced implementation indicates that whole-ward interventions may not always require cognitive engagement from all ward staff to implement changes. Future ward-level implementation studies could consider how best to engage staff and which staff groups to target.

DECLARATIONS

Ethical Approval: Ethical approval for the trial was given by Camden and Kings Research Ethics Committee (reference number: 16/LO/0621) and Scotland Research Ethics Committee A (reference number: 16/SS/0086).

Trial registration number: ISRCTN 99336264.

Funding: This work was supported by NIHR Programme Grants for Applied Research (PGfAR) Programme grant number [ref: DTC-RP-PG-0311-12004].

Contributorship statement

JLC, CF, FP, BP, SPH, and TB made substantial contributions to the conception or design of the work;

SPH, TB and VM-T led on the acquisition of the data;

TB, SPH and JLC led the analysis and interpretation of data for the work;

TB and JLC led the drafting of the paper.

All authors reviewed the paper and gave their final approval of the version to be published;

·All authors give their agreement to be accountable for all aspects of the work in ensuring that questions related to the accuracy or integrity of any part of the work are appropriately investigated and resolved.

There are no competing interests for any author

For peer review only - http://bmjopen.bmj.com/site/about/guidelines.xhtml

Data sharing statement:

Data are available on reasonable request to the corresponding author

REFERENCES

1 World Health Organisation (WHO) *Dementia: a public health priority,* Report: UK 2012. Available from: http://apps.who.int/iris/bitstream/10665/75263/1/9789241564458_eng.pdf?ua=1 (accessed 28 Sep 2018)

2 Gladman J, Porock D, Griffiths A, et al*. Better Mental Health: Care for Older People with Cognitive Impairment in General Hospitals.* Final Report, NIHR Service Delivery and Organisation Programme 2012.

3 Alzheimer's Society. (2009) *Counting the cost*. London: Alzheimer's Society. Available from: https://www.alzheimers.org.uk/sites/default/files/2018-05/Counting_the_cost_report.pdf (accessed 04 Mar 2022).

4 Krupić F, Sadić S, Seffo N, et al. Experience of registered nurses in assessing postoperative pain in hip fracture patients with dementia. *Med Glas (Zenica)* 2018;15:75-80.

5 O'Keeffe S, Lavan J. The Prognostic Significance of Delirium in Older Hospital Patients. *J Am Geriatr Soc* 2015;45(2):174-178.

6 Smith T, Hameed Y, Cross J, *et al.* Assessment of people with cognitive impairment and hip fracture: a systematic review and meta-analysis. *Arch gerontol geriat* 2013;57(2):117-26.

7 Lee HB, Mears SC, Rosenberg PB, *et al.* Predisposing Factors for Postoperative Delirium After Hip Fracture Repair in Individuals with and without Dementia. *J Am Geriatr Soc* 2011;59(12):2306-2313.

8 Seitz DP, Gill SS, Gruneir A, *et al.* Effects of dementia on postoperative outcomes of older adults with hip fractures: a population-based study. *J Am Med Dir Assoc* 2014;15(5):334-41.

For peer review only - http://bmjopen.bmj.com/site/about/guidelines.xhtml

9 National Institute for Health and Care Excellence (NICE). *Hip fracture: management, clinical guideline* [CG124] 2011 updated: 2017 https://www.nice.org.uk/guidance/cg124 (accessed 7 March 2022).

10 Hall AJ, Lang IA, Endacott R. Physiotherapy interventions for people with dementia and a hip fracture—a scoping review of the literature. *Physiotherapy* 2017;103:361-368.

11 Smith T, Yasir AH, Henderson C, et al. Enhanced rehabilitation and care models for adults with dementia following hip fracture surgery (Review). Cochrane Database Syst Rev 2015;6:1-47.

12 Hall AJ, Watkins R, Lang IA, et al. The experiences of physiotherapists treating people with dementia who fracture their hip *BMC Geriatrics* 2017;17:91.

13 Berry SD, Rothbaum RR, Kiel DP, et al. Association of Clinical Outcomes With Surgical Repair of Hip Fracture vs Nonsurgical Management in Nursing Home Residents With Advanced Dementia. *JAMA Intern Med* 2018;178:774–780.

14 Butcher L. Caring for patients with dementia in the acute care setting. *Br J Nurs* 2018;27:358-362.

15 Fogg C, Griffiths P, Meredith P. *et al.* Hospital outcomes of older people with cognitive impairment: An integrative review. *Int J Geriatr Psych*Published Online First: 26 Jun 2018. doi: 10.1002/gps.4919

16 Sampson E, Blanchard M, Jones L, *et al.* Dementia in the acute hospital: Prospective cohort study of prevalence and mortality. *Brit J Psychiat* 2009;195(1):61-66.

17 Gruneir A, Silver MJ, Rochon PA. Emergency department use by older adults: a literature review on trends, appropriateness, and consequences of unmet health care needs. *Med Care Res Rev* 2010;68(2):131-55.

18 Givens JL, Sanft TB, Marcantonio ER. Functional Recovery After Hip Fracture: The Combined Effects of Depressive Symptoms, Cognitive Impairment, and Delirium. *J Am Geriatr Soc* 2008;56:1075-1079.

19 Smith SM, Bayliss EA, Mercer SW, et al. How to design and evaluate interventions to improve outcomes for patients with multimorbidity. *J Comorb* 2013;3:10-17.

20 Hammond SP, Cross J, Shepstone L, et al. Study protocol: Enhancing recovery of patients admitted to acute settings with hip fracture who are identified as experiencing confusion: a multi-centre, cluster-randomised controlled, feasibility trial of the

For peer review only - http://bmjopen.bmj.com/site/about/guidelines.xhtml

PERFECTED Enhanced Recovery (PERFECT-ER) care versus standard acute care. *Trials* 2017;18:583.

21 Richards DA. Complex interventions and the amalgamation of marginal gains: A way forward for understanding and researching essential nursing care? *Int J Nurs Stud* 2015;52(7):1143–1145.

22 Durrand JW, Batterham AM, Danjoux GR. Pre-habilitation (i): aggregation of marginal gains. *Anaesthesia* 2014;69:399–419.

23 Cross JL, Hammond SP, Shepstone L, et al. PERFECTED enhanced recovery pathway (PERFECT-ER) versus standard acute hospital care for people after hip fracture surgery who have cognitive impairment: a feasibility cluster randomised controlled trial. *BMJ Open* 2022;12:e055267.

24 Fox C, Hammond SP, Shepstone L, et al. Development, implementation and a feasibility randomised controlled trial of an enhanced recovery pathway for older people with hip fracture and cognitive impairment in acute hospitals: The PERFECTED applied research programme. *Programme Grants for Applied Research* 2023 [forthcoming].

25 Taylor MJ, McNicholas C, Nicolay C, et al. Systematic review of the application of the plan-do-study-act method to improve quality in healthcare. *BMJ Qual Saf* 2014;23:290-98.

26 Speroff T, O'Conner GT. Study Designs for PDSA Quality Improvement Research. *Qual Manag Health Ca* 2004;13(1):17–32.

27 Haynes AB, Weiser TG, Berry WR, et al. Changes in safety attitude and relationship to decreased postoperative morbidity and mortality following implementation of a checklist-based surgical safety intervention. *BMJ Qual Saf* 2011;20:102-107.

28 Lingard L, Espin S, Rubin B, et al. Getting teams to talk: development and pilot implementation of a checklist to promote interprofessional communication in the OR. *BMJ Qual Saf* 2005;14:340-346.

29 Papoutsi C, Poots A, Clements J, et al. Improving patient safety for older people in acute admissions: implementation of the Frailsafe checklist in 12 hospitals across the UK. *Age Ageing* 2018;47:311-317.

30 Bosk CL, Dixon-Woods M, Goeschel CA, et al. Reality check for checklists. *Lancet* 2009;394:444-5.

For peer review only

For peer review only - http://bmjopen.bmj.com/site/about/guidelines.xhtml

31 Bridges J, May C, Fuller A, et al. Optimising impact and sustainability: a qualitative process evaluation of a complex intervention targeted at compassionate care. *BMJ Qual Saf* 2017;26:970-977.

32 Moore GF, Audrey S, Barker M, et al. Process evaluation of complex interventions: Medical Research Council guidance. *BMJ* 2015;350:h1258.

33 Moore GF, Audrey S, Barker M, et al. *Process evaluation of complex interventions: Medical Research Council guidance.* London: MRC Population Health science Research Network 2014.

34 Langley A, Smallman C, Tsoukas H, et al. Process studies of change in organization and management: unveiling temporality, activity, and flow. *Acad Manage J* 2013;56(1):1-13.

35 Cheng KKF. Metcalfe A. Qualitative Methods and Process Evaluation in Clinical Trials Context: Where to Head to? *IJQM* 2018;17:1-4.

36 Murtagh MJ, Thomson RG, May CR, et al. Qualitative methods in a randomised controlled trial: the role of an integrated qualitative process evaluation in providing evidence to discontinue the intervention in one arm of a trial of a decision support tool. *Qual Saf Health Care* 2007;16:224-229.

37 Sutton E, Herbert G, Burden S, et al. Using the Normalization Process Theory to qualitatively explore sense-making in implementation of the Enhanced Recovery After Surgery programme: "It's not rocket science". *PLoS ONE* 2018;13(4):1-14.

38 Campbell NC, Murray E, Darbyshire J, et al. Designing and evaluating complex interventions to improve health care. *BMJ* 2007;334:455.

39 Campbell M, Fitzpatrick R, Haines A, et al. Framework for design and evaluation of complex interventions to improve health. *BMJ* 2000;321:694-696.

40 Skivington K, Matthews L, Simpson SA, et al. A new Framework for developing and evaluating complex interventions: update of Medical Research Council guidance. BMJ 2021;374:n2061.

41 Skivington K, Matthews L, Simpson SA, et al. Framework for the development and evaluation of complex interventions: gap analysis, workshop and consultation-informed update. *Health Technol Assess* 2021;25(57):1-132.

42 Pinnock H, Barwick M, Carpenter C, et al. Standards for Reporting Implementation Studies (StaRI) statement. *BMJ* 2017;356:i6795

For peer review only - http://bmjopen.bmj.com/site/about/guidelines.xhtml

43 Damschroder LJ, Aron DC, Keith RE, et al. Fostering implementation of health services research findings into practice: a consolidated framework for advancing implementation science. *Implementat Sci* 2009;4(50):1-15.

44 Roberts JL, Pritchard AW, Williams M, et al. Mixed methods process evaluation of an enhanced community-based rehabilitation intervention for elderly patients with hip fracture. *BMJ Open* 2018;8:e021486. doi: 10.1136/bmjopen-2018-021486

45 Szymczak JE. Beyond barriers and facilitators: the central role of practical knowledge and informal network in implementing infection prevention interventions *BMJ Qual Saf* 2018;27:763-765.

46 Sheard L, Marsh C, O'Hara J, et al. Exploring how ward staff engage with the implementation of a patient safety intervention: a UK-based qualitative process evaluation. *BMJ Open* 2017;7:e014558. doi: 10.1136/bmjopen-2016-014558

47 Kummerow Broman K, Kensinger C, Hart H. et al. Closing the loop: a process evaluation of inpatient care team communication. *BMJ Qual Saf* 2015;26:30-32.

For peer review only

Figure and Table Legends

Figure 1: Process Evaluation Data Collection

Table 1: Participant Demographics

Figure 2: PERFECT-ER checklist scores by percentage and implementation site

Figure 3: PERFECTED: Lessons Learned for a Future Definitive Randomised Controlled Trial

Supplementary Figure 1

For peer review only - http://bmjopen.bmj.com/site/about/guidelines.xhtml

	PERFECT-ER checklist scores *n*	SIL action plans *n*	SIL reflective reports *n*	Patient interviews *n*	Relative interviews *n*	Ward staff participants (interviews/ focus groups) *n*
**Ward A**	8	7	21	2	8	10
**Ward B**	8	7	21	0	5	13
**Ward C**	8	6	20	1	5	12
**Ward D**	8	6	15	0	4	15
**Ward E**	8	5	15	0	7	13
**Total**	**40**	**31**	**92**	**3**	**29**	**63**

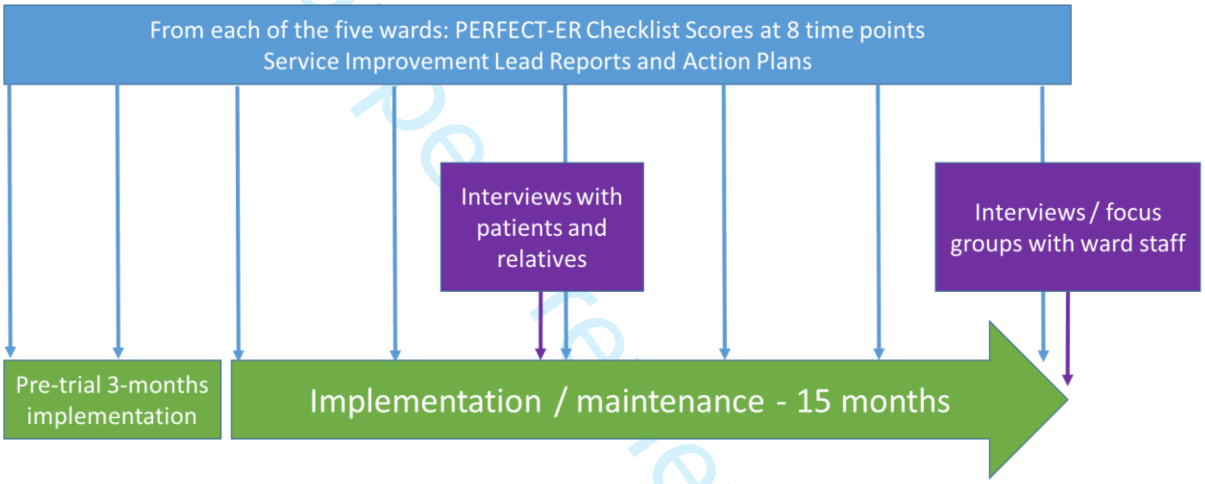

Figure 1: Process Evaluation Data Collection

For peer review only

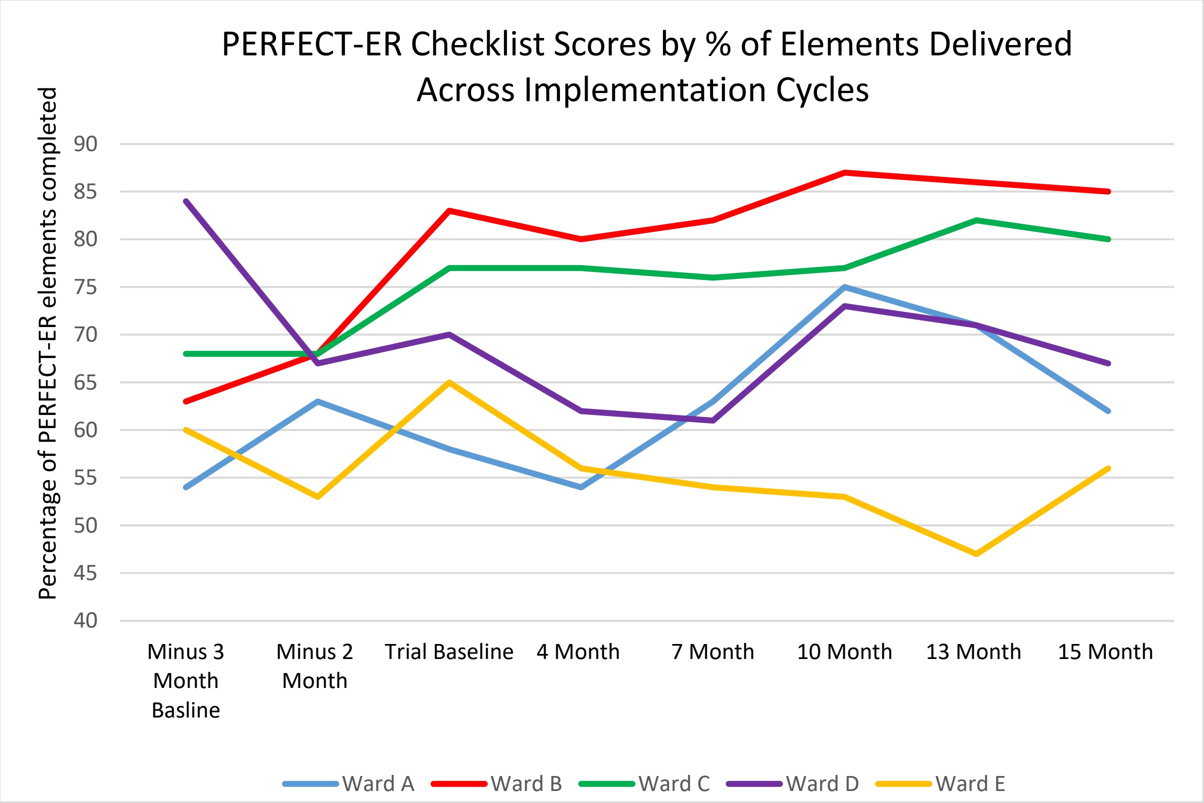

Figure 2: PERFECT-ER checklist scores by percentage and implementation site

For peer review only

PERFECTED Process Evaluation: Lessons Learned

Delivering consistency in intervention

- Protected Service Improvement Lead time must be ensured, consider staff resource when recruiting sites
- If there is more than one Service Improvement Lead at a hospital site, each must have their own responsibilities/accountability
- Service Improvement Leads should be hired from the study ward or more time and support given to those external to the ward
- Consider administrative support for Service Improvement Leads (ward clerk time)
- Create a generic PERFECT-ER leaflet to provide a basic level of information about the intervention for ward staff and provide Service Improvement Lead’s name and contact
- Provide Process Leads with role definition, clear responsibilities and make time allocation transparent
- Refine PERFECT-ER Manual to reflect feedback and role of opinion leaders in implementation

Participant recruitment and retention

- Research team to use a problem solving approach with Process Leads and research nurses to support recruitment and retention of participants
- Provide dementia training for research nurses
- Colour-code participant documents to reduce confusion
- Enable relative participants to fill in Case Report Forms/workbooks in own time rather than at the hospital – possible online option
- Reduce number of outcome measures to decrease burden on relatives and patients
- Consider care-home staff as informants if patients are discharged to a care home and relatives can no longer answer questions about the person’s daily life

Figure 3: PERFECTED: Lessons Learned for a Future Definitive Randomised Controlled Trial

Supplementary Figure 1: PERFECT-ER logic model

Research problem: People with hip fracture and cognitive impairment have poorer outcomes than those without cognitive impairment.

Process outputs

Efficacy outputs

PERFECT-ER Inputs

- SIL .5 for 3 months, then .2 for duration
- SIL 3 day induction training
- PPL .01 per week for 3 months, then .01 per month
- PERFECT-ER Checklist
- PERFECT-ER Manual
- University Staff Member to contact
- Administration / equipment costs

PERFECT-ER Processes and Activities

- SIL completes PERFECT-ER Checklist
- SIL collates scores (Figure 2)
- Ward staff action planning
- SIL works with ward staff to implement PERFECT-ER elements
- Repeat
- SIL raises profile of PERFECT-ER on ward

Changes to Ward Practices

- Adapted practices
- Ward staff using new procedures
- Ward staff documenting actions more
- Standardised practices
- Multiple increased aspects of care such as, carer involvement, more rehabilitation etc

Changes to Ward Ethos

- Embedded re-enablement culture
- Increased staff awareness of the needs of patients with cognitive impairments
- Staff empowerment to make changes and improve care
- Cognitive impairment friendly ward

Staff Impacts & Outcomes

- Staff adapt their practices
- Increased reflective practice
- More / changed work practices
- Staff feel empowered to make changes
- Staff feel part of a stronger team
- Staff demonstrate competence and confidence when looking after cognitively impaired patients

Patient Impacts & Outcomes

- Patients receive better care
- Patients feel better looked after
- Patients have access to more aspects of care
- Patients have better clinical (including 30 day survival) and cognitive outcomes
- Patients have improved quality of life

Carer Impacts & Outcomes

- Carers feel included in and consulted about patient care
- Carers have reduced anxiety about patient care
- Carers know what is happening and what to expect in relation to patient care

Key
SIL: Service Improvement Lead (Generally a qualified staff member)
PL: Process Lead (Consultant level)

Assumptions: Embedding and sustaining numerous small improvements to hip fracture service delivery optimised for patients with CI will standardise care delivery, improving staff confidence and care delivery, leading to improved patient outcomes and patient and carer experience.

External factors that may influence implementation:

- Chronic bed shortages meaning patients on outlier wards (e.g. Winter pressures)
- Chronic staff shortages
- Local organisation policies
- Changes to national guidance

For peer review only

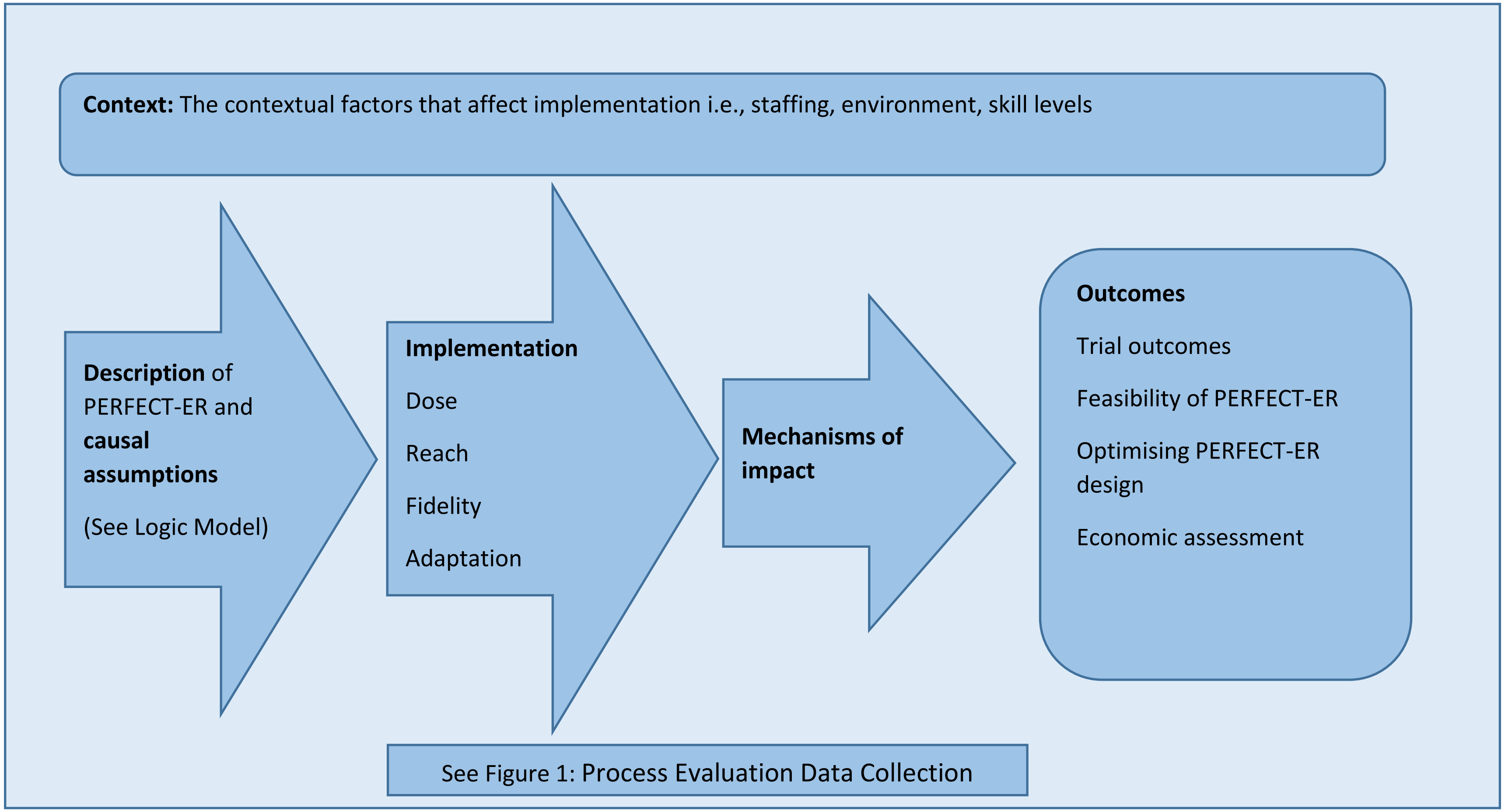
Context: The contextual factors that affect implementation i.e., staffing, environment, skill levels
Description of PERFECT-ER and causal assumptions
(See Logic Model)
Implementation
Dose
Reach
Fidelity
Adaptation
Mechanisms of impact
Outcomes
Trial outcomes
Feasibility of PERFECT-ER
Optimising PERFECT-ER design
Economic assessment
See Figure 1: Process Evaluation Data Collection

Standards for Reporting Implementation Studies: the StaRI checklist for completion

The StaRI standard should be referenced as: Pinnock H, Barwick M, Carpenter C, Eldridge S, Grandes G, Griffiths CJ, Rycroft-Malone J, Meissner P, Murray E, Patel A, Sheikh A, Taylor SJC for the StaRI Group. Standards for Reporting Implementation Studies (StaRI) statement. *BMJ* 2017;356:i6795

The detailed Explanation and Elaboration document, which provides the rationale and exemplar text for all these items is: Pinnock H, Barwick M, Carpenter C, Eldridge S, Grandes G, Griffiths C, Rycroft-Malone J, Meissner P, Murray E, Patel A, Sheikh A, Taylor S, for the StaRI group. Standards for Reporting Implementation Studies (StaRI). Explanation and Elaboration document. *BMJ Open* 2017 2017;7:e013318

Notes: A key concept of the StaRI standards is the dual strands of describing, on the one hand, the implementation strategy and, on the other, the clinical, healthcare, or public health intervention that is being implemented. These strands are represented as two columns in the checklist.

The primary focus of implementation science is the implementation strategy (column 1) and the expectation is that this will always be completed.	The evidence about the impact of the intervention on the targeted population should always be considered (column 2) and either health outcomes reported or robust evidence cited to support a known beneficial effect of the intervention on the health of individuals or populations.

The StaRI standardsrefers to the broad range of study designs employed in implementation science. Authors should refer to other reporting standards for advice on reporting specific methodological features. Conversely, whilst all items are worthy of consideration, not all items will be applicable to, or feasible within every study.

Checklist item		Reported on page #	Implementation Strategy	Reported on page #	Intervention
			"Implementation strategy" refers to how the intervention was implemented		"Intervention" refers to the healthcare or public health intervention that is being implemented.
**Title and abstract**					
Title	**1**	1	Identification as an implementation study, and description of the methodology in the title and/or keywords		
Abstract	**2**	2	Identification as an implementation study, including a description of the implementation strategy to be tested, the evidence-based intervention being implemented, and defining the key implementation and health outcomes.		
**Introduction**					
Introduction	**3**	4	Description of the problem, challenge or deficiency in healthcare or public health that the intervention being implemented aims to address.		
Rationale	**4**	4/5	The scientific background and rationale for the implementation strategy (including any underpinning theory/framework/model, how it is expected to achieve its effects and any pilot work).	5	The scientific background and rationale for the intervention being implemented (including evidence about its effectiveness and how it is expected to achieve its effects).

For peer review only - http://bmjopen.bmj.com/site/about/guidelines.xhtml

Aims and objectives	**5**	5	The aims of the study, differentiating between implementation objectives and any intervention objectives.		
**Methods: description**					
Design	**6**	6-8	The design and key features of the evaluation, (cross referencing to any appropriate methodology reporting standards) and any changes to study protocol, with reasons		
Context	**7**	4-6	The context in which the intervention was implemented. (Consider social, economic, policy, healthcare, organisational barriers and facilitators that might influence implementation elsewhere).		
Targeted 'sites'	**8**	6	The characteristics of the targeted 'site(s)' (e.g locations/personnel/resources etc.) for implementation and any eligibility criteria.	4	The population targeted by the intervention and any eligibility criteria.
Description	**9**	5	A description of the implementation strategy	4-5	A description of the intervention
Sub-groups	**10**	NA	Any sub-groups recruited for additional research tasks, and/or nested studies are described		
**Methods: evaluation**					
Outcomes	**11**	NA Feasibility	Defined pre-specified primary and other outcome(s) of the implementation strategy, and how they were assessed. Document any pre-determined targets	NA Trial paper	Defined pre-specified primary and other outcome(s) of the intervention (if assessed), and how they were assessed. Document any pre-determined targets
Process evaluation	**12**	5	Process evaluation objectives and outcomes related to the mechanism by which the strategy is expected to work		
Economic evaluation	**13**	NA Trial paper	Methods for resource use, costs, economic outcomes and analysis for the implementation strategy	NA Trial paper	Methods for resource use, costs, economic outcomes and analysis for the intervention
Sample size	**14**	7	Rationale for sample sizes (including sample size calculations, budgetary constraints, practical considerations, data saturation, as appropriate)		
Analysis	**15**	8	Methods of analysis (with reasons for that choice)		
Sub-group analyses	**16**	8	Any a priori sub-group analyses (e.g. between different sites in a multicentre study, different clinical or demographic populations), and sub-groups recruited to specific nested research tasks		

For peer review only

For peer review only - http://bmjopen.bmj.com/site/about/guidelines.xhtml

**Results**					
Characteristics	**17**	8-9	Proportion recruited and characteristics of the recipient population for the implementation strategy	Na Trial paper	Proportion recruited and characteristics (if appropriate) of the recipient population for the intervention
Outcomes	**18**	10	Primary and other outcome(s) of the implementation strategy	NA Trial Paper	Primary and other outcome(s) of the Intervention (if assessed)
Process outcomes	**19**	10-16	Process data related to the implementation strategy mapped to the mechanism by which the strategy is expected to work		
Economic evaluation	**20**	NA Trial paper	Resource use, costs, economic outcomes and analysis for the implementation strategy	NA Trial paper	Resource use, costs, economic outcomes and analysis for the intervention
Sub-group analyses	**21**	10	Representativeness and outcomes of subgroups including those recruited to specific research tasks		
Fidelity/ adaptation	**22**	11-13	Fidelity to implementation strategy as planned and adaptation to suit context and preferences	10	Fidelity to delivering the core components of intervention (where measured)
Contextual changes	**23**	13-14	Contextual changes (if any) which may have affected outcomes		
Harms	**24**	NA Trial paper	All important harms or unintended effects in each group		
**Discussion**					
Structured discussion	**25**	16-17	Summary of findings, strengths and limitations, comparisons with other studies, conclusions and implications		
Implications	**26**	16-18	Discussion of policy, practice and/or research implications of the implementation strategy (specifically including scalability)		Discussion of policy, practice and/or research implications of the intervention (specifically including sustainability)
**General**					
Statements	**27**	3	Include statement(s) on regulatory approvals (including, as appropriate, ethical approval, confidential use of routine data, governance approval), trial/study registration (availability of protocol), funding and conflicts of interest		
